# Supplementary material for: Cross-modal integration of metabolomics and cardiac functionality captures dynamic metabotoxic effects of doxorubicin in engineered heart tissues
Source: Stem Cell Reports. 2025 Dec 4;21(1):102725. doi: 10.1016/j.stemcr.2025.102725 (PMC12925965; doi:10.1016/j.stemcr.2025.102725)
Supplement: Document S1. Figures S1–S6, Text S1, and File S1–S4 [file mmc1.pdf]

Stem Cell Reports, Volume *21*

Supplemental Information

Cross-modal integration of metabolomics and cardiac functionality captures dynamic metabotoxic effects of doxorubicin in engineered heart tissues

Federica Conte, Doroteya K. Staykova, Carla Cofiño-Fabres, Danique Snippert, Arno van Rooij, Dirk J. Lefeber, and Robert Passier

Supplemental Data Items

Cross-modal Integration of Metabolomics and Cardiac Functionality Captures Dynamic Metabotoxic Effects of Doxorubicin in Engineered Heart Tissues

Conte, Staykova *et al.* (2025)

Supplemental Items

- **Supplemental Text S1** (page 3): Node enrichment benchmarking.
- **Figure S1** (page 4): 3D-EHT after incubation in metabolite extraction buffer.

  Related to paragraphs "*On-pillar metabolite extraction for sequential functional and metabolic assessment of 3D-EHTs*" and "*On-pillar extraction of polar metabolites*"
- **Figure S2** (page 5): Optic microscope pictures of the 3D-EHTs treated with doxorubicin.

  Related to paragraph "*Doxorubicin reduces contraction force and alters CCM in 3D-EHTs*" and *Figure 2*
- **Figure S3** (page 6): Unfiltered hierarchical cluster plot.

  Related to paragraph "*Node enrichment provides a quantitative measure of association of metabolites with FoC and treatment*" and *Figure 5*
- **Figure S4** (page 7): Significance analysis via standard statistics of TEA-based metabolomics dataset (Z-score boxplots).

  Related to paragraph "*Metabolomics data processing*"
- **Figure S5** (page 8): Significance analysis via standard statistics of TBA-based metabolomics dataset (Z-score boxplots).

  Related to paragraph "*Metabolomics data processing*"
- **Figure S6** (page 9): Significance analysis via standard statistics of AA-based metabolomics dataset (Z-score boxplots).

  Related to paragraph "*Metabolomics data processing*"

Supplemental data

The following supplemental files (1-4) and the original code have been deposited at deposited at FigShare.com, under the DOI: https://doi.org/10.6084/m9.figshare.27985364.v2, and are publicly available at as of the date of publication.

- **Supplemental File S1**: List of MS transitions of target metabolites used for data acquisition.

- **Supplemental File S2**: Samples' metabolite levels (normalized and Z-transformed), dsDNA content and metadata.
- **Supplemental File S3**: Interactive topological connectivity network (TCN).
- **Supplemental File S4**: Data matrix with node values across the entire TCN used for correlation analysis.
- **Original data processing code (Python)** used for TDA-guided integration and visualization.

Raw data files have been deposited in the FAIR-compliant repository MetaboLights, under the accession number MTBLS13225, and are publicly available as of the date of publication.

Supplemental Text S1. Node enrichment benchmarking

After constructing the TCN, sample subsets from each node were extracted. Nodes sharing edges contained overlapping samples. For each cluster, mean metabolite values were computed and defined as node values (or coordinates) in the metabolic space. For categorical metadata (e.g., treatment, timepoint, or their combination), node values represented the percentage of samples with the respective category. These node values were assembled into a new matrix spanning the entire network (**Supplemental File 4**), from which a Spearman rank correlation matrix was calculated to estimate associations between data and metadata.

To benchmark our de novo method for inferring relationships of interest via node-based calculations, we evaluated the correlations between FoC and the metabolites. Being a numerical-valued parameter, FoC enabled the estimation of Spearman's rank correlation coefficients in two ways, namely (i) via the Z-scores and (ii) via the node values associated with each metabolite, referred as ground-truth and de novo correlations, respectively. Through plotting the latter as coordinates in a scatter plot, we visually compared the correlation coefficients obtained by both methods and estimated how the node-based coefficients approximated the ground-truth ones (**Figures 5A-D**).

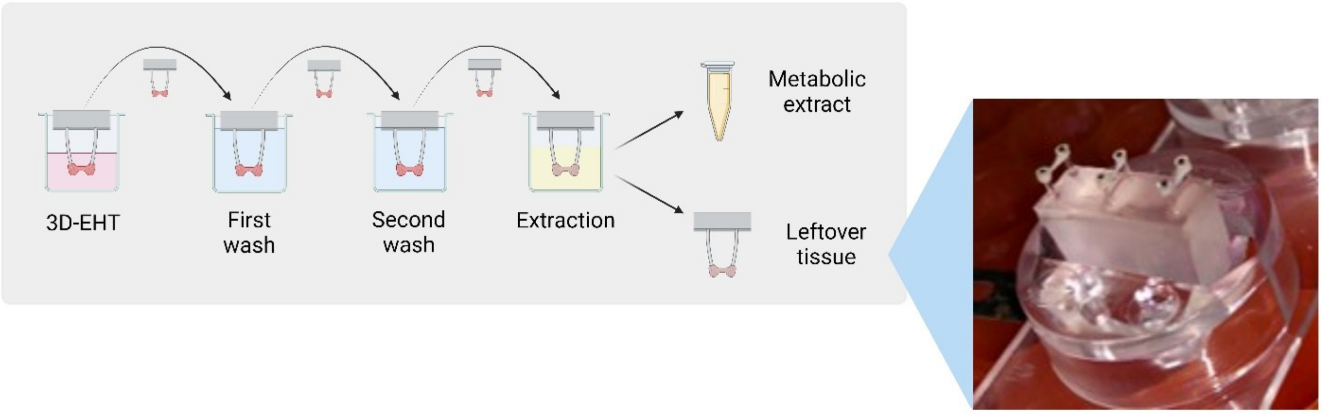

Figure S1.

3D-EHT tissues after 10 minutes incubation in organic extraction buffer during on-pillar extraction. With the correct handling, our extraction method ensures the preservation of the tissue integrity for later use (e.g. dsDNA extraction). Note: the picture illustrates a PDMS holder with three pairs of pillars that was used, however for our study, the design of the holder was

customized to one pillar set only, to enable on-pillar extraction from single tissues.

Treatment		*Replicate 1*	*Replicate 2*	*Replicate 3*
Untreated	0 hours	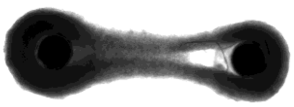	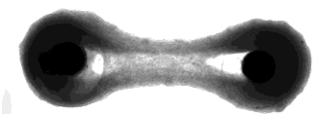	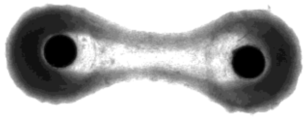
DMSO	24 hours	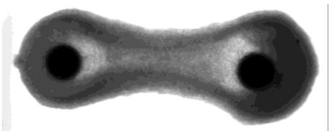	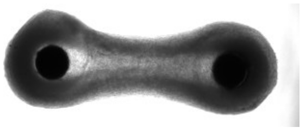	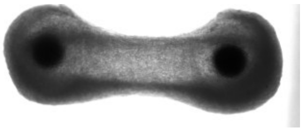
DOXO 1 uM	24 hours	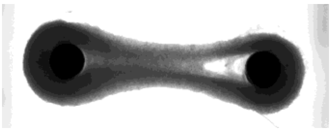	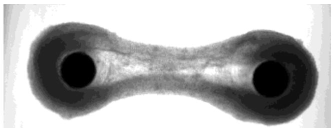	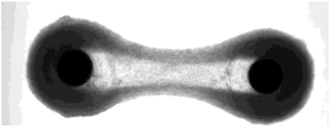
DOXO 5 uM	24 hours	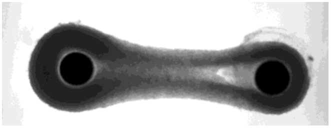	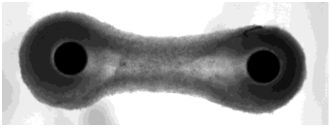	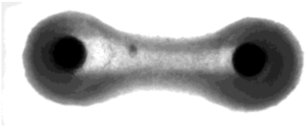
DMSO	48 hours	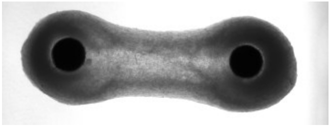	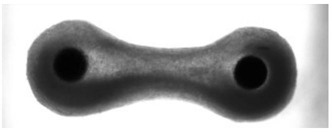	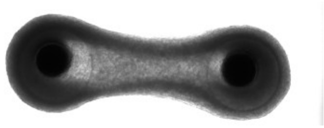
DOXO 1 uM	48 hours	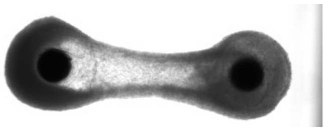	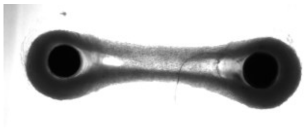	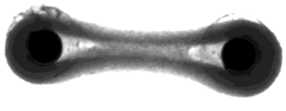
DOXO 5 uM	48 hours	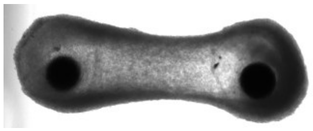	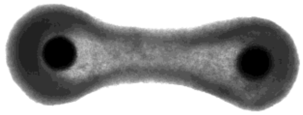	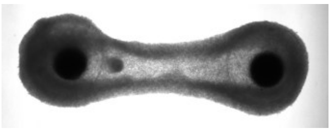

Figure S2.

Pictures of all 3D-EHT replicates acquired after electrical pacing and immediately before FoC measurement and metabolite extraction using a Nikon Ti2-E inverted microscope. The pictures were acquired when each tissue was at maximum extension (relaxed state). Distance between pillars' heads: 3 mm.

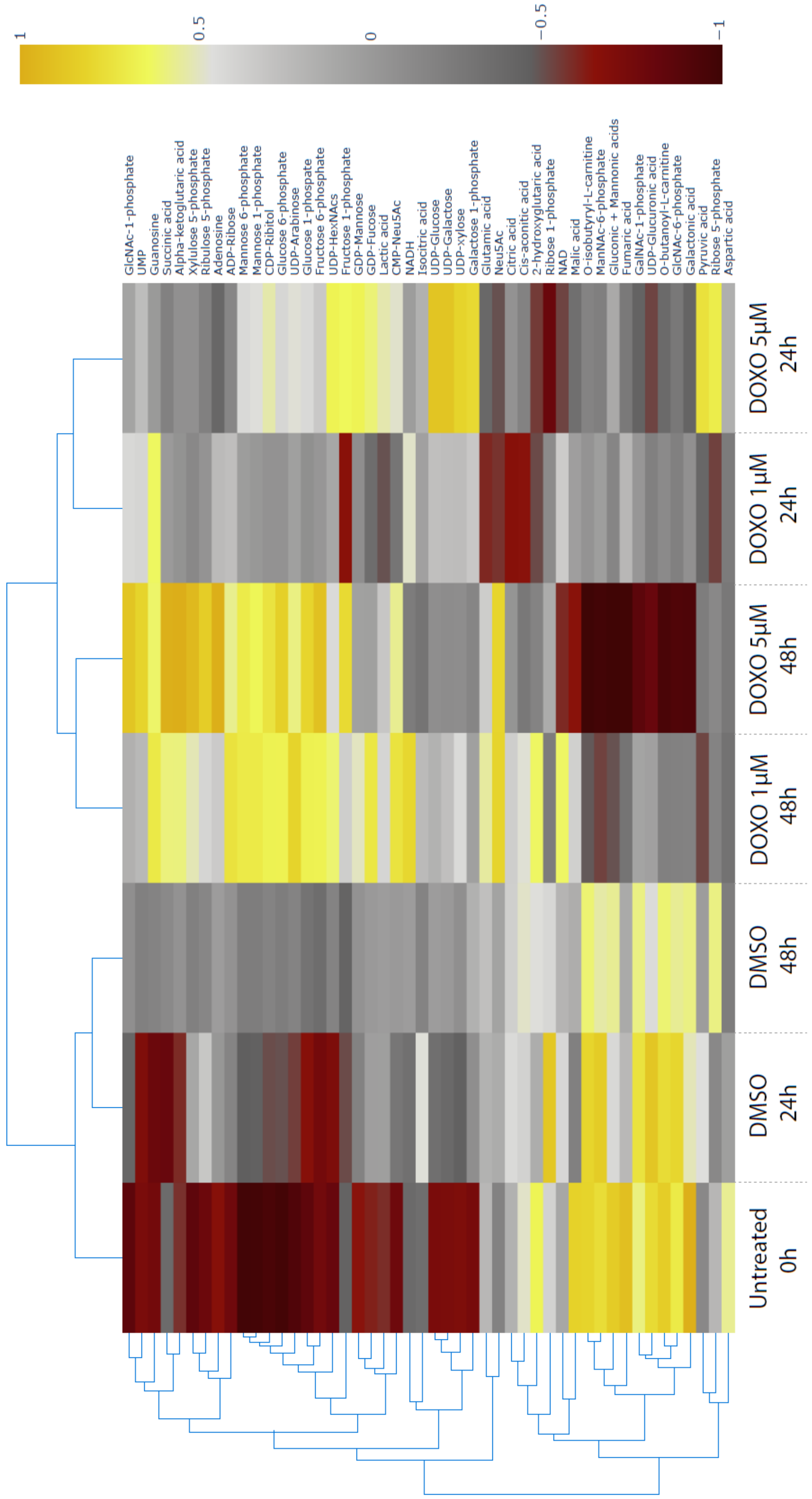

Figure S3.

Heatmap with dendrograms of hierarchical clustering on metabolites and treatment-time pairs based on their Spearman's rank correlation coefficients prior to filtering. For more details see main text.

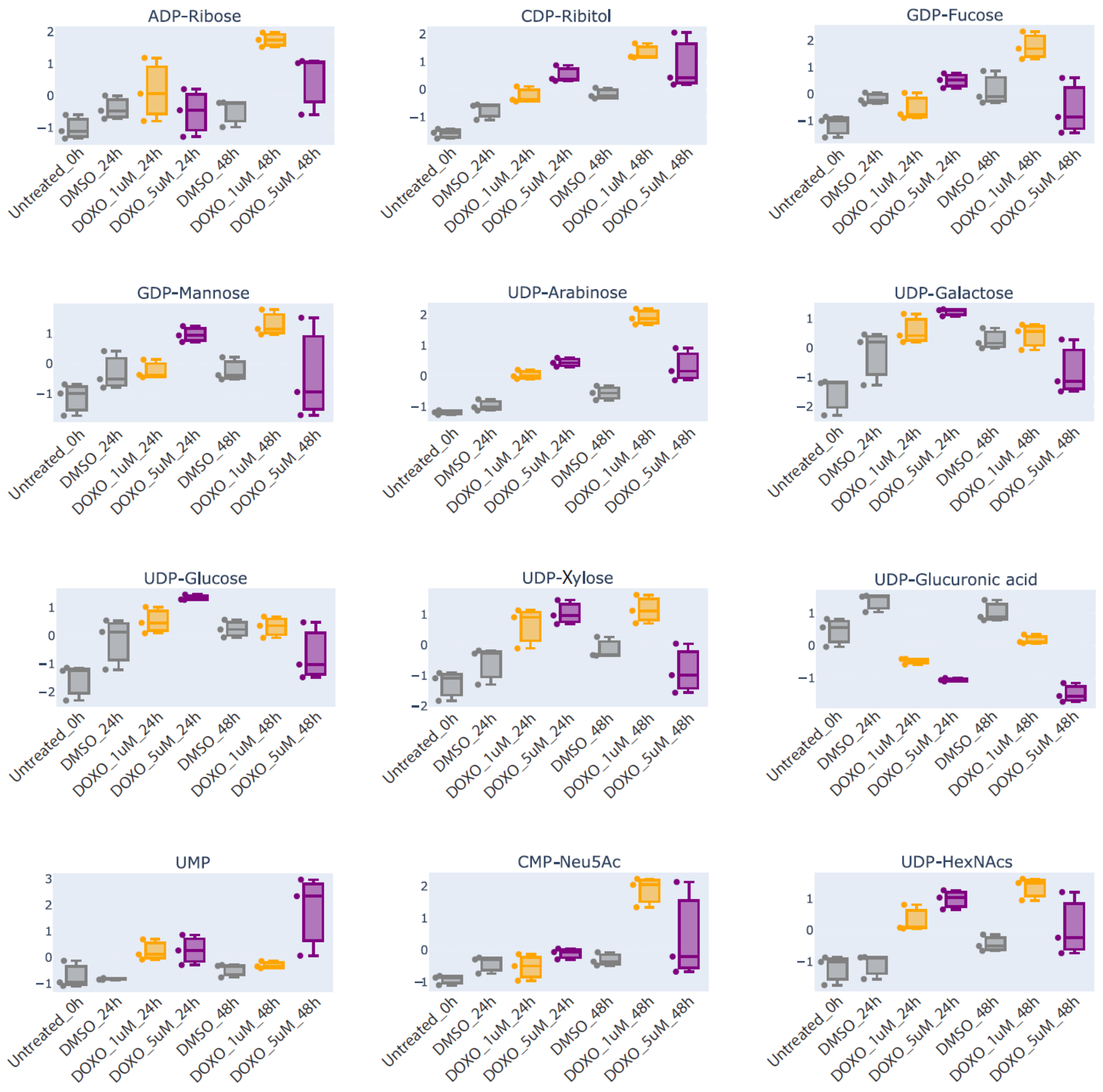

Figure S4.

Boxplots of the Z-transformed normalized levels of the metabolites detected via TEA-based MS method. Abbreviations: see main figures.

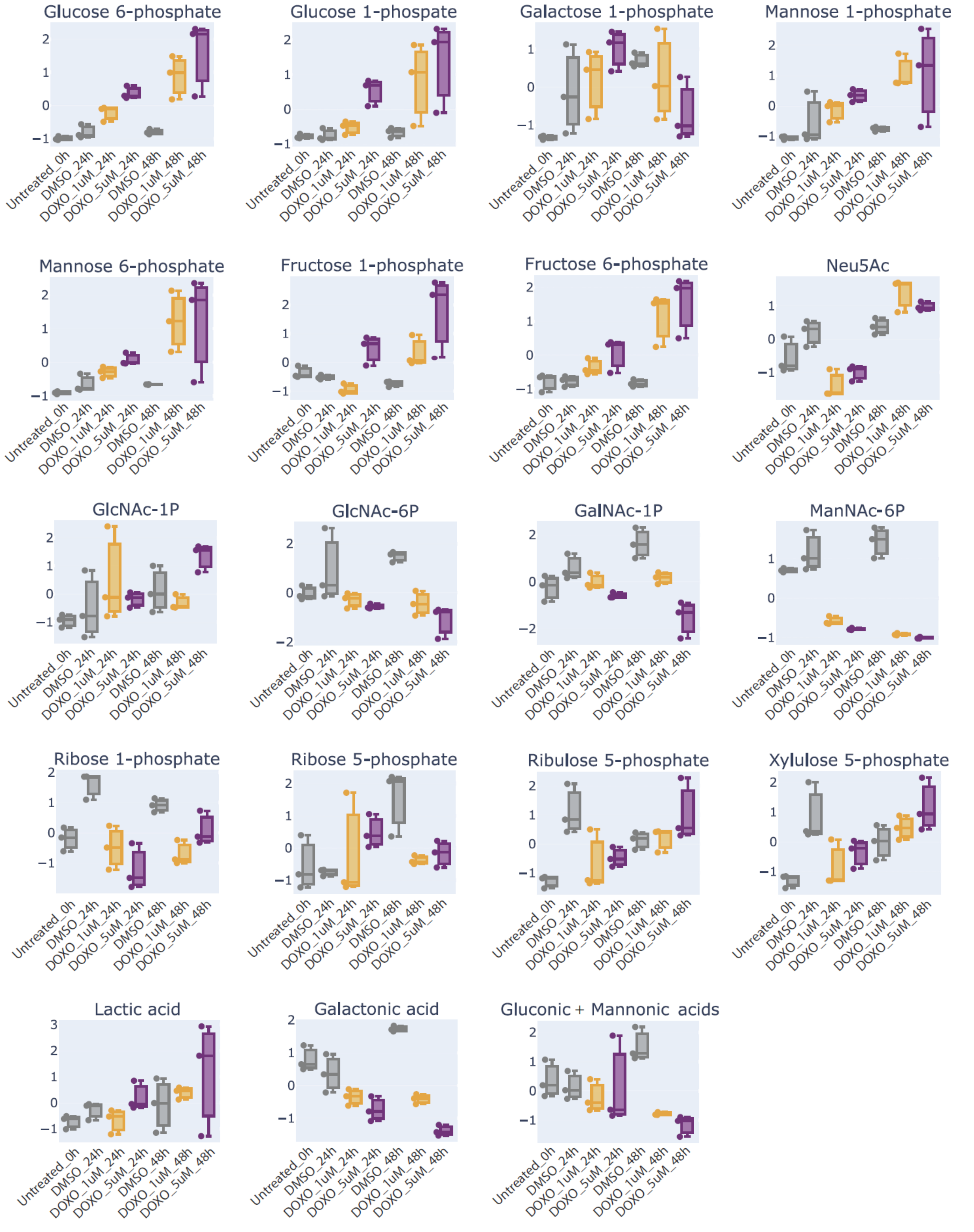

Figure S5.
Boxplots of the Z-transformed normalized levels of the metabolites detected via TBA-based MS method. Abbreviations: see main figures.

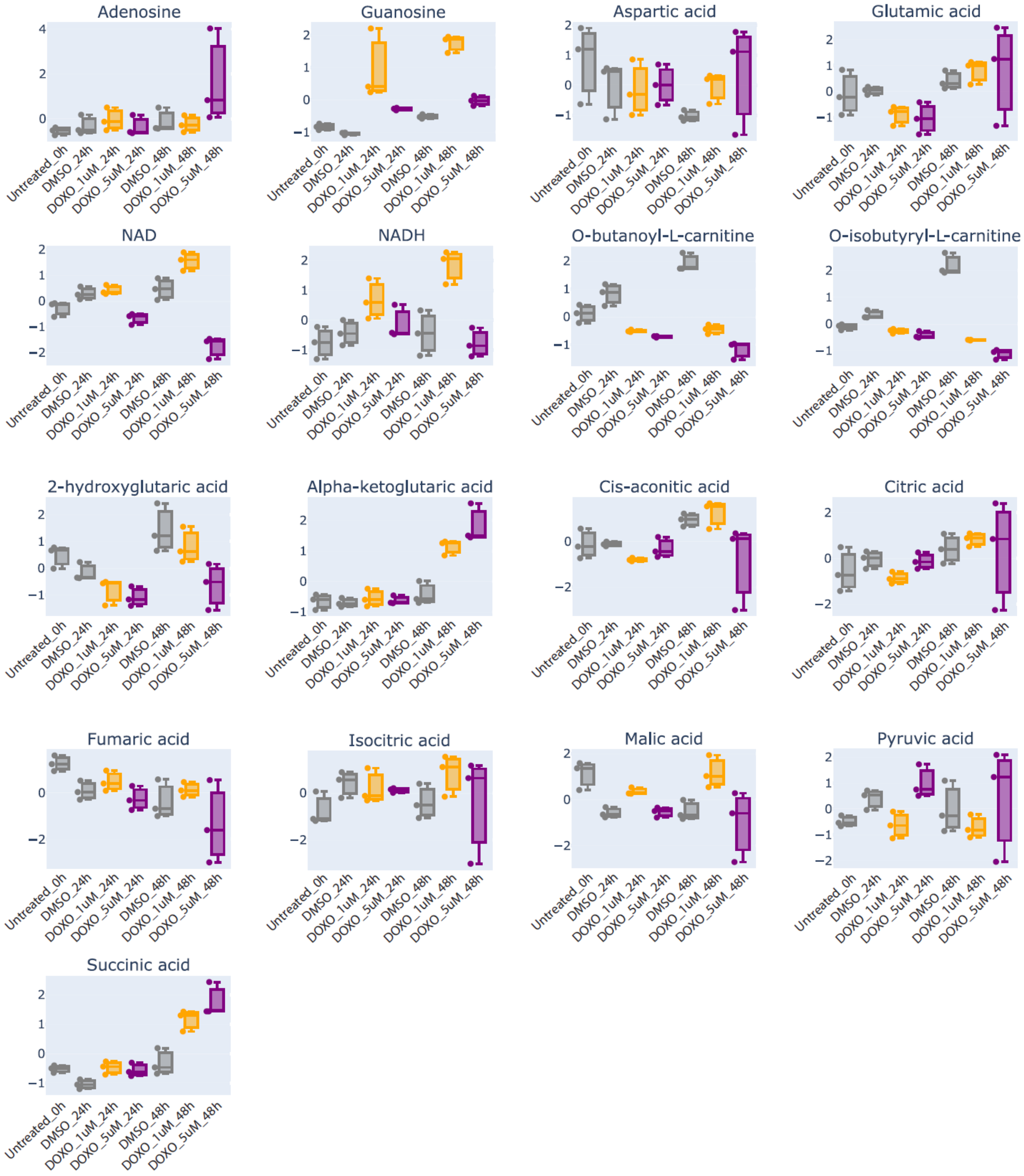

Figure S6.

Boxplots of the Z-transformed normalized levels of the metabolites detected via AA-based MS method. Abbreviations: see main figures.
