# Supplementary material for: Cross-modal integration of metabolomics and cardiac functionality captures dynamic metabotoxic effects of doxorubicin in engineered heart tissues
Source: Stem Cell Reports. 2025 Dec 4;21(1):102725. doi: 10.1016/j.stemcr.2025.102725 (PMC12925965; doi:10.1016/j.stemcr.2025.102725)
Supplement: Document S2. Article plus supplemental information [file mmc2.pdf]

STEM CELL REPORTS
Article

ISSCR

OPEN ACCESS

Cross-modal integration of metabolomics and cardiac functionality captures dynamic metabotoxic effects of doxorubicin in engineered heart tissues

Federica Conte,[1,2,3,7] Doroteya K. Staykova,[1,4,5,7] Carla Cofiño-Fabres,[2] Danique Snippert,[2] Arno van Rooij,[1] Dirk J. Lefeber,[1,3,4,8,*] and Robert Passier[2,6,8,9,*]

[1]Department of Human Genetics, Translational Metabolic Laboratory (TML), Radboud University Medical Center, 6525GA Nijmegen, the Netherlands
[2]Applied Stem Cell Technologies Group, Department of Bioengineering Technologies, TechMed Centre, University of Twente, 7522NB Enschede, the Netherlands
[3]Department of Neurology, Donders Institute for Brain, Cognition and Behavior, Radboud University Medical Center, 6525GA Nijmegen, the Netherlands
[4]United for Metabolic Diseases, National Infrastructure for Metabolic Disease Research, the Netherlands
[5]Multicore Dynamics Ltd, New Milton, UK
[6]Department of Anatomy and Embryology, Leiden University Medical Centre, 2311EZ Leiden, the Netherlands
[7]These authors contributed equally
[8]Senior author
[9]Lead contact
*Correspondence: Dirk.Lefeber@radboudumc.nl (D.J.L.), robert.passier@utwente.nl (R.P.)
https://doi.org/10.1016/j.stemcr.2025.102725

SUMMARY

Functional stem cell-derived heart models offer new avenues for preclinical, animal-free physiological assessment of drug cardiotoxicity. Yet, comprehensive molecular profiling in these models remains limited, leaving key metabolic drivers of cardiotoxicity unexplored. Here, we leveraged an innovative platform and a topology-guided integration framework to unveil the complex dose- and time-dependent metabolic rewiring of the central carbon metabolism caused by doxorubicin-induced cardiotoxicity (DiC) in human heart tissue. Through cross-modal integration of cardiac functionality and metabolomics in 3D engineered heart tissues, we identified 20 metabolites linked to cardiac contraction and differentially affected by doxorubicin exposure. Nine of them, including carnitine esters and uridine 5′-diphosphate (UDP)-glucuronic acid, were never before implicated in DiC and may represent promising candidates for DiC metabolic rescue. By yielding high-resolution insights into complex biological mechanisms, our platform and mathematical framework enable metabolic and functional assessment of cardiotoxicity in engineered heart models, paving the way for innovative advances in preclinical drug development.

INTRODUCTION

Drug development is a lengthy and exceedingly costly process, which requires 10–15 years and up to \$2.8 billion per molecule on average (Schlander et al., 2021; Wouters et al., 2020). Including the preclinical stage, the failure rate of drug development exceeds 90%, primarily due to insufficient clinical efficacy in humans (40%–50%) and the occurrence of unmanageable toxicity (30%), which may arise during development or even post-marketing (Dirnagl et al., 2022; Onakpoya et al., 2016, 2018; Sun et al., 2022). Emblematic is the case of doxorubicin (DOXO), a potent anticancer drug with high effectiveness against several types of solid tumors and hematologic malignancies (Sritharan and Sivalingam, 2021). Despite its efficacy, the clinical applications of DOXO have been severely limited due to its toxic effects, which include progressive and irreversible cardiotoxicity (Avagimyan et al., 2022; Henriksen, 2018; Mitry and Edwards, 2016; Renu et al., 2018; Sawyer, 2013; Volkova and Russell, 2011; Zamorano et al., 2016). Cardiac symptoms linked to DOXO-induced cardiotoxicity (DiC) range from echocardiographic anomalies (up to 50% of treated patients) (Robinson et al., 2023) and sub-clinical cardiac dysfunction (up to 17.9% of treated patients) (Lotrionte et al., 2013) to severe cardiomyopathy and congestive heart failure (up to 9% of treated patients) (Linders et al., 2024). Nonetheless, the pathophysiological mechanisms underlying DiC remain controversial and incompletely understood (Bordoni et al., 1999; Bugger et al., 2011; Carvalho et al., 2010; Mordente et al., 2009; Pugazhendhi et al., 2018; Renu et al., 2018; Robinson et al., 2023; Singal and Iliskovic, 1998; Strigun et al., 2012). More effective preclinical trials could help identify cardiotoxic effects prior to clinical evaluation, while reducing time, cost, and risks associated with drug development. Yet, the lack of predictive preclinical models to evaluate drug efficacy and cardiotoxicity in humans has been a major obstacle (Oh et al., 2019). Preclinical cardiac research has traditionally relied on animal models (*in vivo*), cardiac tissues (*ex vivo*), and cardiac cell cultures (*in vitro*). Although animal models offer detailed insights into functional and molecular mechanisms during development and disease, their use is limited by ethical concerns, high costs, need of dedicated infrastructures, and interspecies pathophysiological differences that compromise result reliability and translatability to human medicine (Greek and Menache, 2013; Milani-Nejad and Janssen, 2014; Pound and Ritskes-Hoitinga, 2018;

© 2025 The Authors.
Published by Elsevier Inc. on behalf of International Society for Stem Cell Research.
This is an open access article under the CC BY license (http://creativecommons.org/licenses/by/4.0/).

Shin et al., 2021; Van Norman, 2019; Zaragoza et al., 2011). Similarly, *ex vivo* samples of human cardiac tissue come with their own challenges (Oh et al., 2019; Zhou et al., 2022), including ethical concerns, limited availability, and scarcity of healthy control tissues, complicating the design of scientifically robust comparative studies. On the other hand, human 2D cardiac cell cultures, either primary cardiomyocytes (CMs) or human stem cell -derived CMs, are very informative but fall short in recapitulating the complexity, functionality, and pathophysiology of the heart (Ahuja et al., 2004; Guo and Pu, 2020; Voigt et al., 2015). However, recent advancements in cardiac tissue engineering have enabled the generation of *in vitro* 3D cardiac models that recapitulate functional aspects of the cardiac tissue, while remaining of human origin.

Although functional 3D cardiac models offer new possibilities for preclinical physiological studies, in-depth molecular characterization through advanced mass spectrometry-based omics (MS-omics), such as metabolomics, is still lacking in these models. Yet, integrating physiological and molecular data could greatly advance the preclinical understanding of drug metabolism and cardiotoxicity, broadening the use of these cardiac models for drug assessment before clinical trials.

The integration of functional and molecular data from functional 3D cardiac models is hindered by several technical challenges. As the generation of these models is often time-consuming, costly and requires dedicated expertise and equipment, the resulting experiments are typically affected by low statistical power (low sample size, limited replicate number) and high noise. Besides, downstream applications of high-throughput omics technologies produce large, heterogeneous data affected by strong dimensional imbalance (low number of samples but high number of features measured per sample). This makes downstream data analysis especially challenging (Kaur et al., 2021) as these aspects often violate the assumptions required for the application of mainstream statistical methods (Hoekstra et al., 2012; Thiese et al., 2015). Moreover, standard statistics imposes constraints when it comes to the integration of data across different modalities (e.g., metabolic data and functional data), hampering the extraction of actionable biological insights. Altogether, these aspects render standard statistics inadequate for delivering robust results in experiments combining molecular and functional investigations. Alternative mathematical techniques that facilitate data integration and interpretation can help circumvent the heterogeneous, noisy, and dimensionally imbalanced nature of molecular and functional data obtained from 3D model-based experiments (Ebbels et al., 2023). A new set of topological-based methods, broadly referred to as topological data analysis (TDA), is rapidly evolving in the biomedical field (Skaf and Laubenbacher, 2022) thanks to its unique ability to integrate different data types and elucidate underlying structure of complex data. This is particularly important for biomedical applications in this data-driven era, as it enables the integration of different types of information (molecular, functional, and even clinical) and the identification of complex biological mechanisms (Amézquita et al., 2020), thus revolutionizing the process of data exploration, hypothesis generation, and knowledge extraction.

In this study, we propose an innovative platform and the first mathematical framework for cross-modal integration of functional and metabolic data to explore the metabolic alterations underlying cardiotoxicity in human cardiac tissue. First, we developed a novel protocol for sequential force of contraction (FoC) assessment and MS-based metabolomics profiling from 3D engineered heart tissues (3D-EHTs) (Ribeiro et al., 2022) treated with DOXO. Next, we devised and benchmarked an innovative TDA-based framework for cross-modal integration of functional and metabolic data, which revealed for the first time in a human cardiac model the complex metabolic rewiring of the central carbon metabolism (CCM) differentially triggered by DOXO in a time- and dose-dependent manner. Our study offers a paradigm shift in the integration of cross-modal data and provides proof of concept of how the incorporation of different layers of information enhances the molecular understanding of complex biological processes in functional tissue models, which is critical for effective preclinical drug development and toxicity assessment.

RESULTS

On-pillar metabolite extraction for sequential functional and metabolic assessment of 3D-EHTs

To enable sequential functional and metabolic assessments on each single 3D-EHT, while limiting tissue manipulation to prevent breakage and metabolic alterations, we developed an on-pillar extraction method for polar metabolites that directly follows the video-based FoC recording. Recently, we established a novel platform for the generation and functional evaluation of human induced pluripotent stem cell (hiPSC)-derived EHTs formed around flexible polydimethylsiloxane (PDMS) pillars (Ribeiro et al., 2022), which facilitates the deflection of the pillars in response to the contractile force exerted by the EHT. For this study, we adapted the design of this EHT platform by reducing the number of tissues per holder from three to one (Figure 1A), allowing the collection of metabolites from each single tissue after FoC was recorded. Upon optimization, we determined that two washing steps with 4 mL of washing buffer were sufficient to ensure the full removal

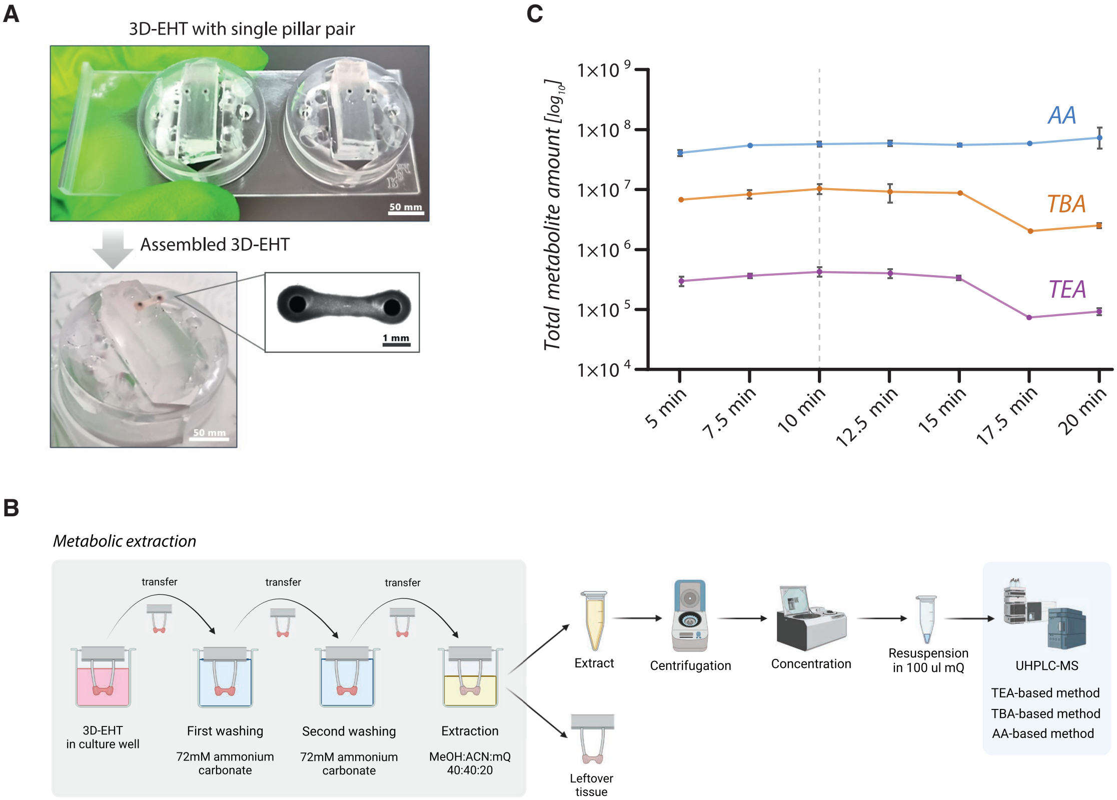

Figure 1. Optimization of metabolite extraction from 3D engineered heart tissues (3D-EHTs)

(A) Bottom view of a 3D-EHT holder with single pair of PDMS pillars, stained with black carbon for subsequent image analysis, before (upper panel) and after (lower panel) tissue casting.

(B) Schematic workflow of the optimized on-pillar extraction of polar metabolites from 3D-EHTs. Abbreviations: ACN, acetonitrile; AA, acetic acid-based method; MeOH, methanol; mQ, MS-compatible milli-Q water; TBA, tributylamine-based method; TEA, triethylamine-based method; UHPLC-MS, ultra-high-performance liquid chromatography-mass spectrometry.

(C) Evaluation of increasing incubation times in extraction buffer to identify the minimum duration required to achieve maximal metabolite yield across the three targeted metabolomics methods. Black bars indicate standard deviation. Each point represents the average of three 3D-EHTs ($n = 3$).

of the culture medium from the tissue and PDMS holder, which could be a potential source of contamination for downstream metabolomics analysis (Figure S1). Increasing incubation times in extraction buffer were tested and identified 10 min as the minimum duration yielding maximal metabolite recovery (Figures 1B and 1C). The resulting on-pillar extraction protocol is illustrated in Figure 1B and described in the *Methods* section. After extraction, the holder with the tissue (Figure S1) was removed, and the extraction buffer, now containing metabolites, was collected, spun down, and concentrated overnight, prior to resuspension in ultra-pure water for targeted metabolomics analysis (Figure 1B). In total, 48 metabolites linked to the CCM and energy homeostasis were successfully detected and quantified via three targeted liquid chromatography-mass spectrometry (LC-MS) methods, namely triethylamine (TEA)-based method, tributylamine (TBA)-based method, and acetic acid (AA)-based method (Figures 1B and 1C, File S1).

Our simple and fast on-pillar extraction protocol allows us to minimize tissue manipulation and handling-time before extraction, while reducing the risk of artificial metabolic changes and sample-prep variability, which would compromise data quality and interpretation.

DOXO reduces contraction force and alters CCM in 3D-EHTs

Once the protocol for sequential functional and metabolic assessment was optimized, we assessed the effects of DOXO on the functionality (FoC) and metabolism (CCM) of 3D-EHTs.

After 13-day differentiation of hiPSCs to CMs (Rivera-Arbeláez et al., 2022), differentiated CMs were metabolically selected through glucose starvation and lactate

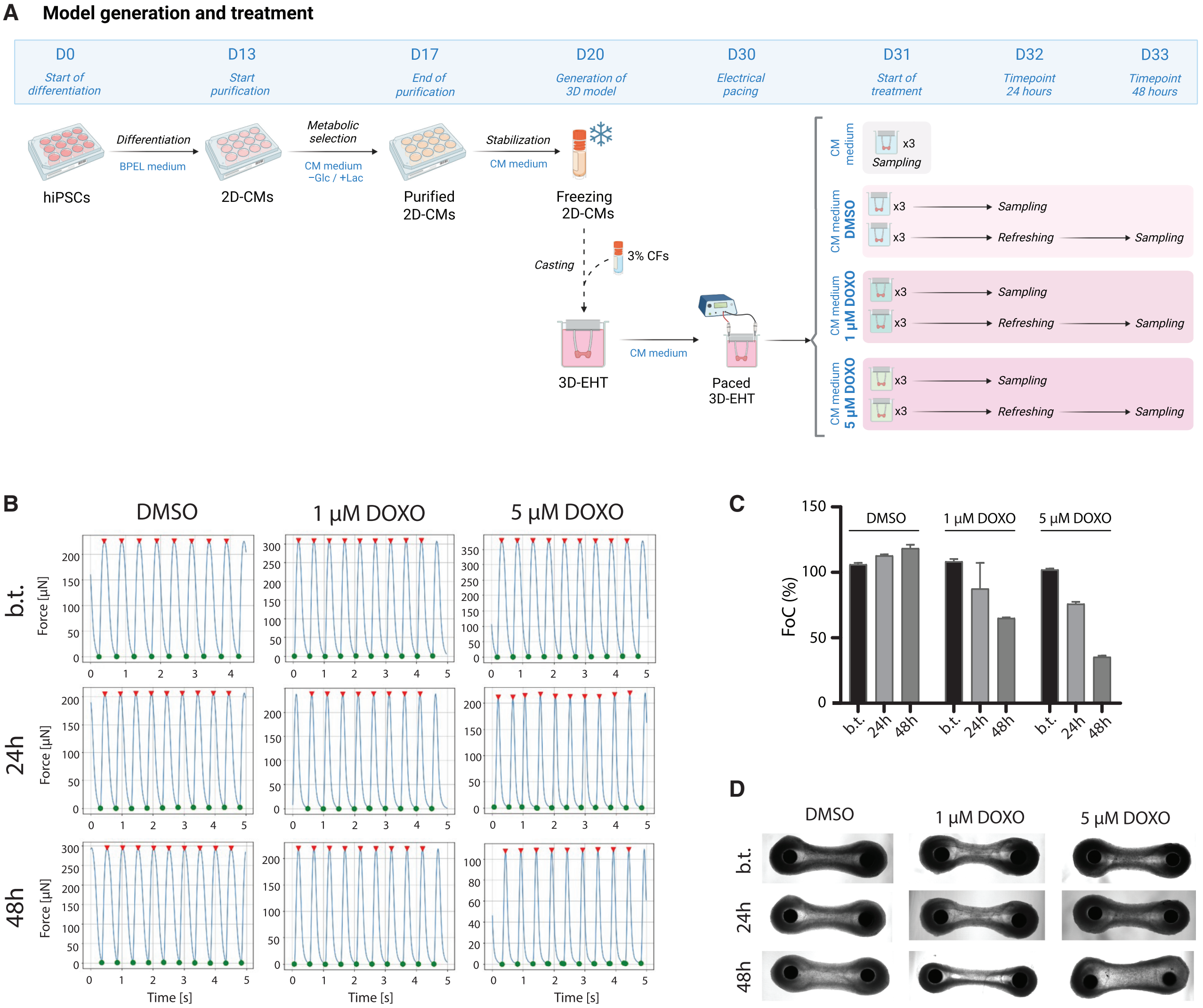

Figure 2. Workflow of the doxorubicin treatment and force of contraction response

(A) Workflow of the 3D-EHT generation (D0–20) and electrical pacing, followed by DOXO treatment. Abbreviations: 2D, two-dimensional; 3D, three-dimensional; b.t., before treatment; D, day; CM, cardiomyocytes; CF, cardiac fibroblasts; DMSO, dimethyl sulfoxide; DOXO, doxorubicin; EHT, engineered heart tissue; Glc, glucose; Lac, lactate.

(B) Graphs showing the representative force overtime from one (out of three) replicate for each condition. The tissues were electrically paced during video recording (2 Hz) prior to any treatment, as previously described (Rivera-Arbeláez et al., 2022). *y* axis: force expressed in micronewton (µN); *x* axis: time expressed in seconds (s).

(C) Quantification of the force of contraction (FoC), plotted as percentage means (variation represents standard error of the mean for $n=3$).

(D) Pictures of one (out of three) 3D-EHT replicate for each condition/time point. Distance between pillars' heads: 3 mm. The pictures of all replicates taken prior to FoC measurement and metabolic extraction are reported in Figure S2.

feeding (days 13–17), recovered for 3 days (days 17–20), and then cryopreserved (Figure 2A). Only batches with more than 90% cardiac troponin T (cTnT)-positive CMs were used for EHT generation (Ribeiro et al., 2022). To generate 3D-EHTs, 2.45×10^5 CMs were mixed with 3% of human cardiac fibroblasts (CFs) and with matrix components to enable the creation of the tissue between the PDMS pillars' heads. The resulting 3D-EHTs were cultured for ten days (days 20–30), during which the CMs aligned and reorganized to form spontaneously contracting cardiac tissues. On day 31, the EHTs for time point 0 (baseline, without treatment) were recorded for FoC followed by metabolite extraction, while the EHTs designated for collection at subsequent time points were refreshed with medium containing either DMSO (control condition) or 1 or 5 µM DOXO (Schwach et al., 2024) (Figure 2A), and incubated for 24 h

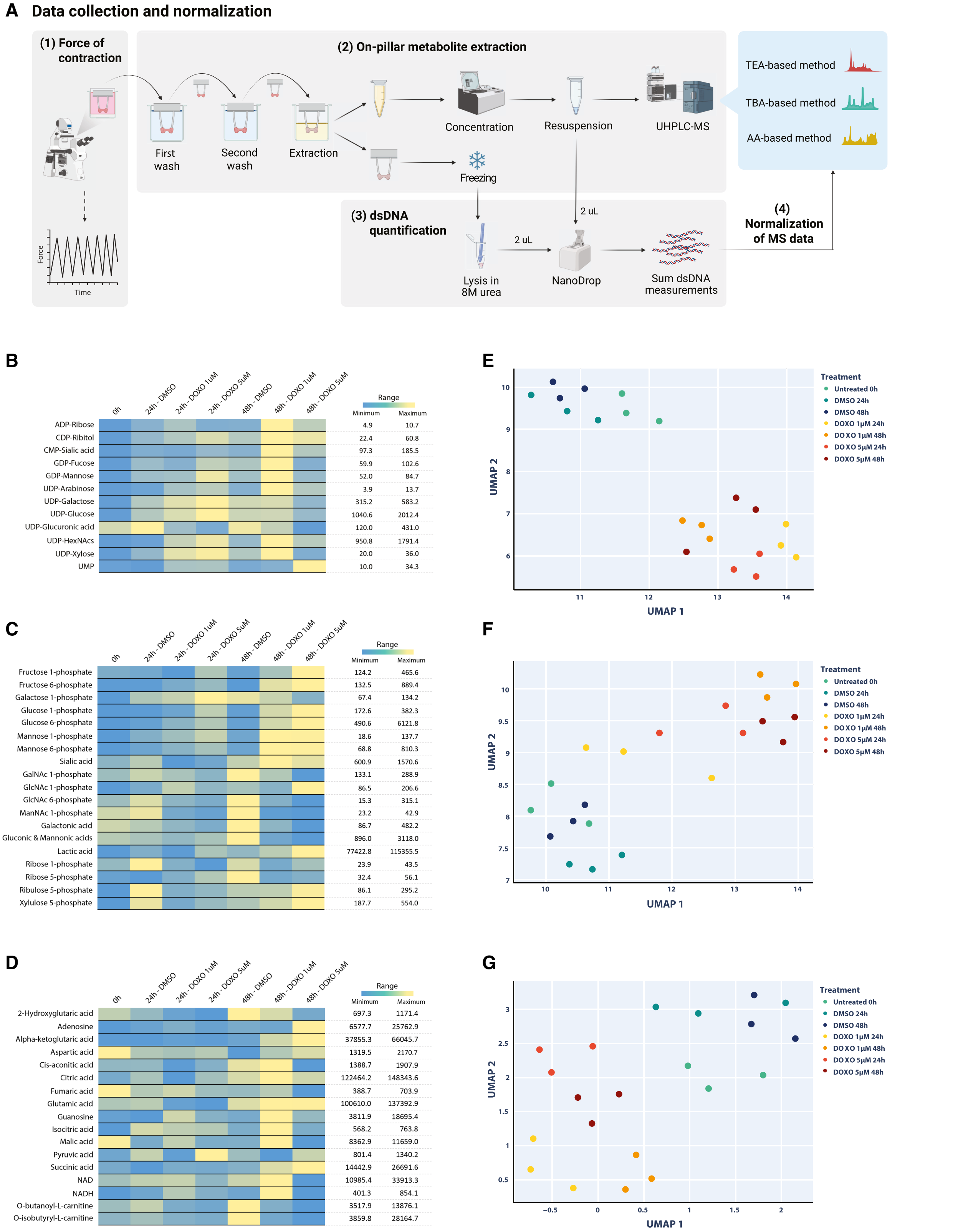

(legend on next page)

(day 32) or 48 h (day 33), before undergoing FoC recording and metabolite extraction. These DOXO concentrations (1 and 5 μM) align with doses commonly applied in *in vitro* cardiotoxicity tests (Schwach et al., 2024), as these levels induce consistent functional impairment and fall within the plasma concentration range observed in patients (Barpe et al., 2010).

Upon morphological inspection, 3D-EHTs did not show differences in morphology and integrity caused by DOXO treatments (Figures 2D and S2). Instead, from a functional standpoint, FoC analysis of control tissues (treated with DMSO) revealed an increase of FoC over time, while tissues treated with DOXO showed a progressive reduction in FoC during the same time window. The reduction in FoC was comparable between the two doses at 24 h but became more pronounced at 48 h for tissues treated with 5 μM DOXO (Figures 2C and 2D). Immediately after FoC recording, on-pillar extraction of polar metabolites was performed for each tissue (Figure 3A), and the resulting extracts were later used for metabolomics analysis. Metabolomics data were normalized based on dsDNA content measured both in the metabolic extract and in the leftover tissue upon lysis in 8 M urea (Figure 3A, File S2).

Through the TEA-based method, we found that most nucleotide sugars were elevated in treated 3D-EHTs, especially those treated for 24 h with 1 μM DOXO, when compared to untreated tissues (Figure 3B). The only exception was uridine 5′-diphosphate (UDP)-glucuronic acid, which conversely showed high levels in control tissues at all timepoints and low levels in tissue treated with the highest DOXO dose (Figure 3B). Using the TBA-based method, we found that hexose phosphates and sialic acid reached their highest levels in 3D-EHTs treated for 48 h, either with 1 or 5 μM DOXO (Figure 3C). Hexosamine phosphates, instead, were markedly higher in untreated tissues, with the only exception of *N*-acetyl-glucosamine 1-phosphate (GlcNAc-1P). In fact, GlcNAc-1P, along with lactate, showed the highest levels in tissues treated with 5 μM for 48 h (Figure 3C). Pentose phosphates displayed a less clear trend, which appeared more time- than dose-dependent (Figure 3C). Lastly, using the AA-based method, we detected patterns of intensity varying with treatment and time. Adenosine, aspartate, glutamate, alpha-ketoglutarate, citrate, pyruvate, and succinate showed their highest levels in 3D-EHTs treated for 48 h with 5 μM DOXO. The opposite pattern was instead shown by fumarate and malate, which reached the highest levels in control tissues at 0 h (Figure 3D). The rest of the metabolites showed peak levels in tissues treated with 1 μM for 48 h, except for the two carnitine esters, which reached the maximum in controls (Figure 3D).

To investigate the sample distribution and clustering based on their metabolic profiles, we applied non-linear dimensionality reduction via uniform manifold approximation and projection (UMAP) on Z-transformed normalized data (Figures 3E–3G). In each UMAP projection, the data from the control tissues cultured in DMSO clustered in the same area, regardless of the collection time point. Likewise, the samples treated with 1 and 5 μM DOXO clustered together in the UMAP projection from the TEA-based method (Figure 3E). However, they displayed more separation in the projection resulted from the TBA-based method (Figure 3F) and AA-based method (Figure 3G).

TDA reveals sample association based on contraction force, treatment dose and time

To infer metabolic dynamics of DiC in 3D-EHTs, we aimed to explore relationships between individual layers of information (metabolic profile, FoC, treatment dose and time) through cross-modal integration. To overcome the challenges of integrating heterogeneous, high-dimensional data derived from limited sample size with inter-batch variability, we singled out TDA as the best fit-for-purpose approach. TDA is a mathematical framework that provides a set of topological tools to explore and infer relevant features from highly complex data, overcoming unavoidable limitations posed by mainstream standard statistics (Chazal and Michel, 2021). The most popular TDA algorithm, called *Mapper*, employs algebraic topology to produce a graph, also known as topological connectivity network (TCN), able to reveal the underlying shape (topological structure) of complex integrated data (Lum et al., 2013; Singh et al., 2007).

In our study, we applied the *Mapper* algorithm with two main purposes: to reveal the topological structure of the metabolomics data as seen through the topological lenses of UMAP (Figures 4E–4G) and to execute a *de novo* protocol for cross-modal integration of functional and metabolomics data (Equations 1, 2, 3, and 4; Figures 4A–4D). The resulting TCN revealed two distinct groups of connected nodes (topological clusters), indicated with *A* and *B* (Figure 4B), also referred to as connected components

Figure 3. Metabolomics results of 3D-EHTs treated with doxorubicin
(A) Schematic workflow of the data collection and on-pillar metabolite extraction. For abbreviations, see captions of Figures 1 and 2.
(B–D) Heatmaps representing the normalized levels of each metabolite detected across the sample set from TEA analysis (B), TBA analysis (C), and AA analysis (D).
(E–G) Two-component UMAP embedding obtained for TEA dataset (E), TBA dataset (F), and AA dataset (G) using correlation distance metric calculated from metabolite *Z* scores.

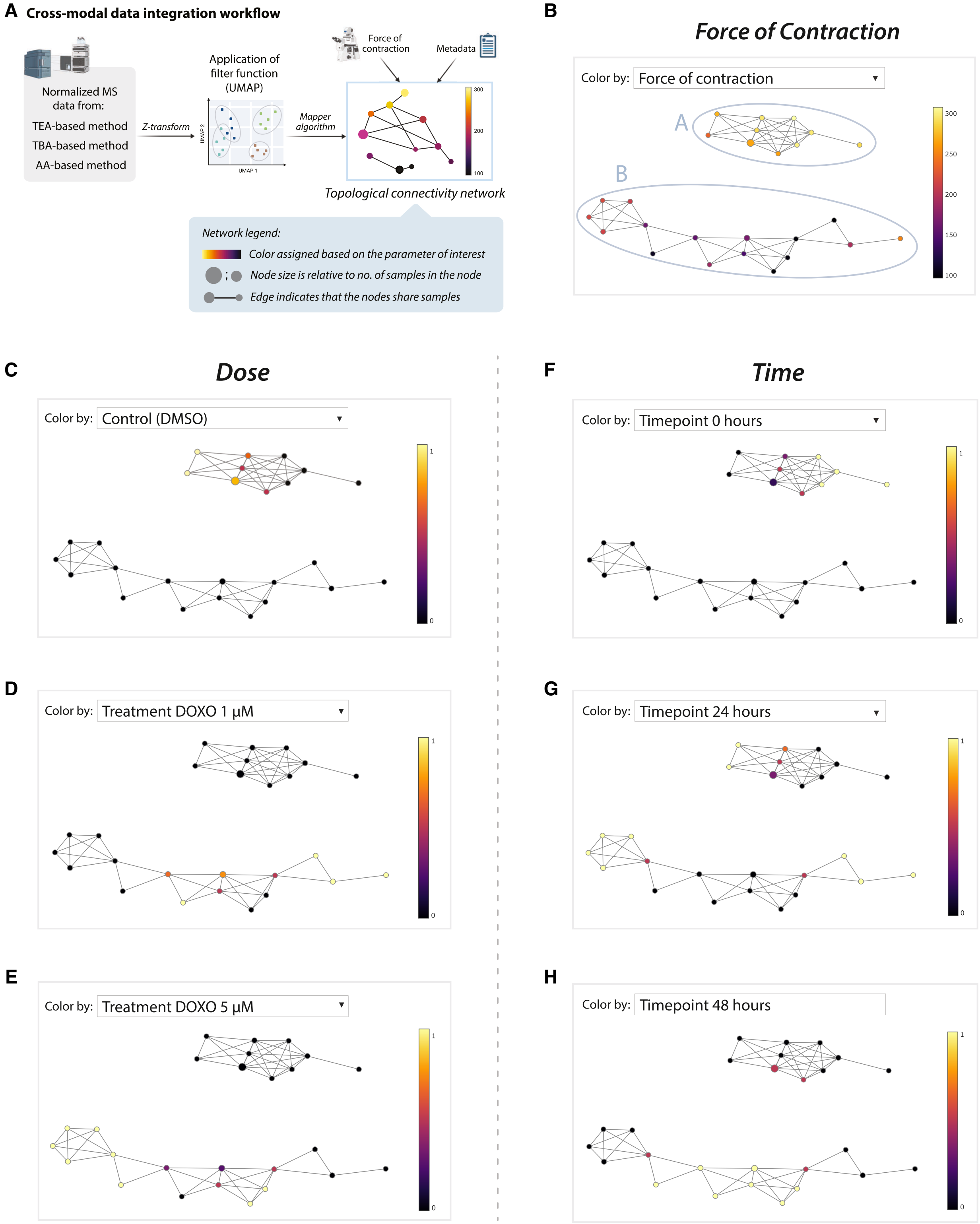

Figure 4. TDA-based data processing and resulting topological connectivity network

(A) Schematic workflow of the data processing and integration via *Mapper* algorithm (*giotto-tda*) (Tauzin et al., 2021). TCN with three lenses (UMAP projections of TEA, TBA, and AA sets, Figures 3E–3G) revealed two connected components (A and B) with nodes (sample

(legend continued on next page)

(Chazal and Michel, 2021). The first connected component (*A*) is composed of ten nodes and shows one lateral branch of nodes, referred to as a flare (Carrière et al., 2018; Joshi and Joshi, 2020), while the second component (*B*) is formed by 16 nodes and two lateral flares. Based on the edges drawn between nodes that have one or more samples in common, the nodes of component *A* revealed high connectivity, indicating high similarity in the metabolic profiles of corresponding samples. In contrast, the nodes in component *B* exhibited less connectivity and an elongated arrangement, which points to a greater variability in the metabolomics profiles of the associated samples.

By using the upper dropdown menu in the interactive TCN file (File S3), the nodes can be colored based on metadata information incorporated as staining parameters, such as numerical metadata (e.g., FoC, Figure 4B), categorical metadata (e.g., treatment type, Figures 4C–4E, or time point; Figures 4F–4H) and by metabolic levels of each individual metabolite (File S2).

When coloring the TCN based on FoC (Figure 4B), all the samples with highest FoC localize in component A, while those with lower FoC are concentrated in component B (Figure 4B). Based on molecular data alone, the two components of TCN model were able to discriminate samples with varying FoC values. Similarly, when coloring the nodes based on treatment type, we found that control samples (utreated and treated with DMSO) were clustered in the nodes of component A (Figure 4C), while the samples treated with DOXO (both doses) appeared in the nodes of component B (Figures 4D and 4E), confirming separation also based on treatment type. Lastly, when coloring nodes based on the time point of collection, the samples collected at time point 0 (non-treated) appeared only in component A, specifically in the lateral flare (Figure 4F). Samples collected at 24 h appeared both in component A (EHTs treated with DMSO, controls) and in component B (EHTs treated with 1 μM DOXO in the right flare, tissues treated with 5 μM in the left flare). With two flares associated with low and high treatment dose response (Figures 4D and 4E), TCN revealed a high diversity in samples treated with DOXO after 24 h, inferring dose-related molecular changes.

Lastly, samples collected at 48 h appeared in component A (EHTs treated with DMSO, controls) and some in the central (core) nodes of component B (Figure 4H). The latter suggests that at this time point the EHTs treated with both DOXO doses exhibited higher similarity. Patterns of associations between the levels of a given metabolite and FoC, treatment dose, or time can be qualitatively evaluated by comparing the coloring of the TCN nodes and verifying whether the resulting patterns match (File S3).

Current integration approaches predominantly operate at the feature level, analyzing each omics readout separately before comparing significant features to identify potential links or enriched pathways (Subramanian et al., 2020; Yuan et al., 2020). On the contrary, our TDA-based framework allows mathematical cross-modal integration at a data level, by using samples as anchors to bridge different layers of information while maximizing the knowledge extraction from each individual layer. Moreover, this framework allows us to visually explore the topological shape of the connectivity existing among the samples through the TCN, a unique attribute of topology. Here, we exploited the proposed mathematical pipeline to integrate data from three different targeted metabolomics methods with FoC, and generated an interactive TCN that displays clear separation between samples based on FoC, treatment dose and time.

Translating categorical metadata into quantitative information via topological node enrichment

As already demonstrated, TCN enabled qualitative analysis through the visual exploration of sample clusters, represented by nodes, and their relationship with both types of metadata, categorical (e.g., treatment type, exposure time) and numerical (FoC). To translate this qualitative information into quantitative data that can be analyzed via standard statistics, we developed a new approach based on topological node enrichment (TNE). TNE starts with the calculation of the mean value within each node, based on the values that the samples included in the node display for a certain varying modality (e.g., a metabolite, FoC, or other parameters of interest, either numerical or categorical). Next, the mean values calculated across the nodes of the entire network enabled the transition from a complex metric of categorical metadata to quantitative (numerical) values (Figure 5A). The latter allows for subjecting varying types of metadata to mainstream statistical analysis. Specifically, in our study we employed Spearman's rank correlation coefficients to estimate the strength of relationships between metabolomics *Z* scores and metadata of interest (e.g., FoC) within the node-based framework.

To demonstrate the reliability and robustness of our novel approach, we performed an unbiased comparison

clusters) stained according to their mean FoC (B) or fraction of samples with associated treatment (C–E) or time point (F–H), ranging between 0 (no clustered samples) and 1 (all clustered samples). The connected component A is formed by nodes including control samples treated with DMSO collected at all three time points (with time point 0 located in the lateral flare). The connected component B is formed by nodes including samples treated with DOXO, both 1 and 5 μM, with those collected at 24 h located in the two lateral flares, and those collected at 48 h localized in the core nodes of the component. The interactive version of the TCN is provided as Supplemental File 3.

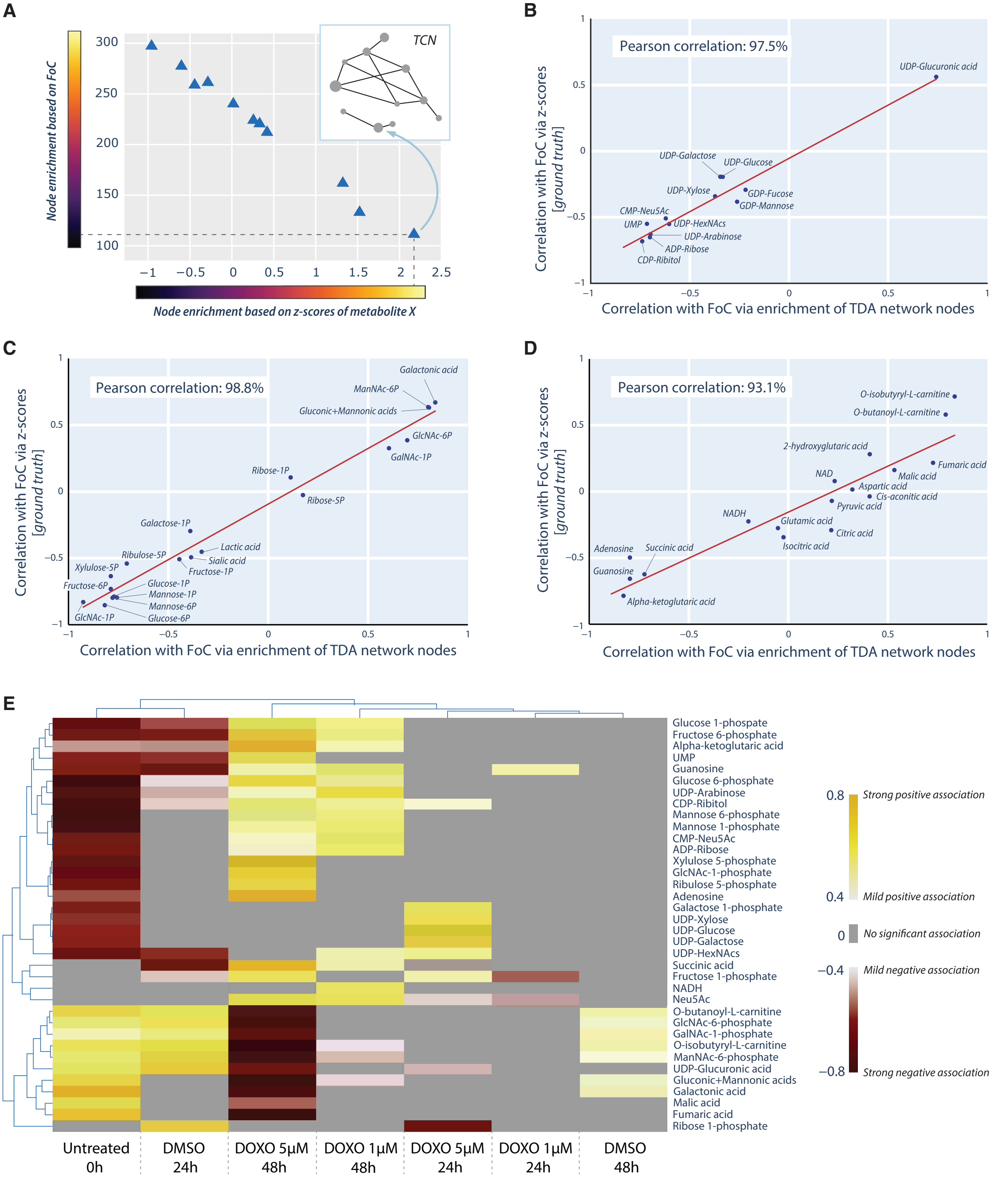

Figure 5. Correlation based on topological node enrichment (TNE) as quantitative measure of association between each metabolite and DOXO treatment dose and time

(A) Theoretical example of TNE definition, leveraging the coloring of node based on force of contraction (numerical metadata) and the levels of a hypothetical metabolite (X).

(legend continued on next page)

between our TNE-based approach and a second approach independent from the TDA framework. This test was possible thanks to the fact that the FoC was considered a numerical metadata and thus was not used to create the network model, which was instead only driven by the metabolomics data. As a result, this offered the unique opportunity to use FoC as an independent mathematical anchor to perform the comparison of two independent approaches for the calculation of association. The first approach consisted in calculating the correlation between each metabolite and the FoC (also referred to as *ground-truth*), independently from the TDA framework. The second approach instead was based on the calculation of correlation using TNE (Figure 5A). Ground-truth and TNE-based estimates of associations with the FoC were calculated for each metabolite derived from each separate metabolomics dataset. Resulting pairs of Spearman's rank correlation coefficients for all metabolites were displayed as scatterplots (Figures 5B–5D). All three datasets showed strong linear relationships with Pearson correlation coefficients above 90%, validating the ability of node enrichment-based correlations to capture relationships between different data modalities. The latter is of particular importance for categorical or binary metadata where ground-truth calculations are not a viable option.

In conclusion, TNE approach offers an elegant solution to an outstanding challenge of integrating multi-omics with non-omics data and metadata (López de Maturana et al., 2019), with potential implications in biomedical research at large.

Node enrichment provides a quantitative measure of association of metabolites with FoC and treatment

To quantitatively map the correlation of all metabolites with treatment dose and time in a single plot, we performed hierarchical clustering (HC) analysis of Spearman's rank correlation coefficients using Dash Bio (Hossain, 2019) (Figure S3). To highlight the most relevant associating metabolites, a 3-step data filtering based on correlation values and p values was applied, resulting in 36 metabolites with mild-to-strong association (positive or negative) with treatment dose and time (Figure 5E). To describe data points collected in a time sequence, we consider DOXO dose and exposure time as two independent axes of a Cartesian system, in which the location of a point can be described via two values (coordinates) represented by the dose and exposure time, indicated as (*dose,time*) pair. The correlation values obtained via our framework need to be interpreted in respect to the *(dose,time)* pair, which described the state of our tissue model in the dose-time continuum. For sake of simplicity, in this paper we refer to (*dose,time*) pairs as dose at exposure time.

From the HC plot (Figure 5E), opposite patterns in control EHTs versus EHTs treated with DOXO emerged clearly. Specifically, untreated EHTs at 0 and DMSO-treated EHTs at 24 h show negative correlating metabolites in the upper part of the HC plot (red) and positively correlating metabolites in the bottom part (yellow). DOXO treated EHTs instead showed the opposite association pattern with increasing clarity when moving toward the highest dose and longest exposure time. We assessed how the strength of association of each metabolite changes with dose and time using HC of correlation values. To visualize the results in the context of the CCM, we represented the detected mildly-to-strongly associating metabolites from the three most extreme conditions (0 h, baseline; 48 h, 1 μM DOXO; 48 h, 5 μM DOXO) in metabolic maps, where they remained colored based on the correlation values of the HC plot (Figures 6A–6C). These snapshots enabled us to highlight how certain groups of metabolites associate with different DOXO doses and exposure time. For example, hexose phosphates (glucose-/mannose-/fructose-6-phosphate, glucose-/galactose-/mannose-/fructose-1-phosphate) and pentose phosphates (ribose-/ribulose-/xylulose-5-phosphate) exhibit a strong negative association with untreated EHTs at 0 h (Figure 6A), while showed strong positive association with 1 μM DOXO at 48 h (Figure 6B) and even stronger positive association with 5 μM DOXO at 48 h (Figure 6C). On the contrary, metabolites linked to the aerobic respiration and mitochondrial TCA (such as fumarate, malate and carnitine esters), showed strong positive association with untreated ETHs 0 h, but mild negative association in 1 μM DOXO at 48 h and strong negative association in tissues treated with 5 μM DOXO at 48 h. Nucleotide sugars resulted mostly positively associated with DOXO treatments and negatively with untreated EHTs at 0 h, with the only exception of UDP-glucuronic acid, which displayed the opposite trend. Similarly to UDP-glucuronic acid, also aldonic acid sugars, galactonic, gluconic and mannonic acids, showed

(B–D) Scatter plots showing the relationship between (i) Spearman's rank correlation coefficients calculated using metabolite Z scores and FoC and (ii) coefficients based on TCN nodes (via TNE) for each dataset (B, TEA dataset; C, TBA dataset; D, AA dataset).

(E) A Dash Bio clustergram displaying hierarchical clustering of metabolites and (*dose,time*) pairs based on their Spearman's rank correlation coefficients, after a 3-step filtering. The unfiltered clustergram is available as Figure S3. The lowest linkages within the row dendrogram (left side) indicate groups of metabolites that exhibit similar patterns of associations with (*dose,time*) pairs. The latter are also arranged according to the similarity of corresponding samples (column dendrogram, top side). Yellow color indicates (mild-to-strong) positive association between the metabolite and the (*dose,time*) pairs, while red color indicates (mild-to-strong) negative association.

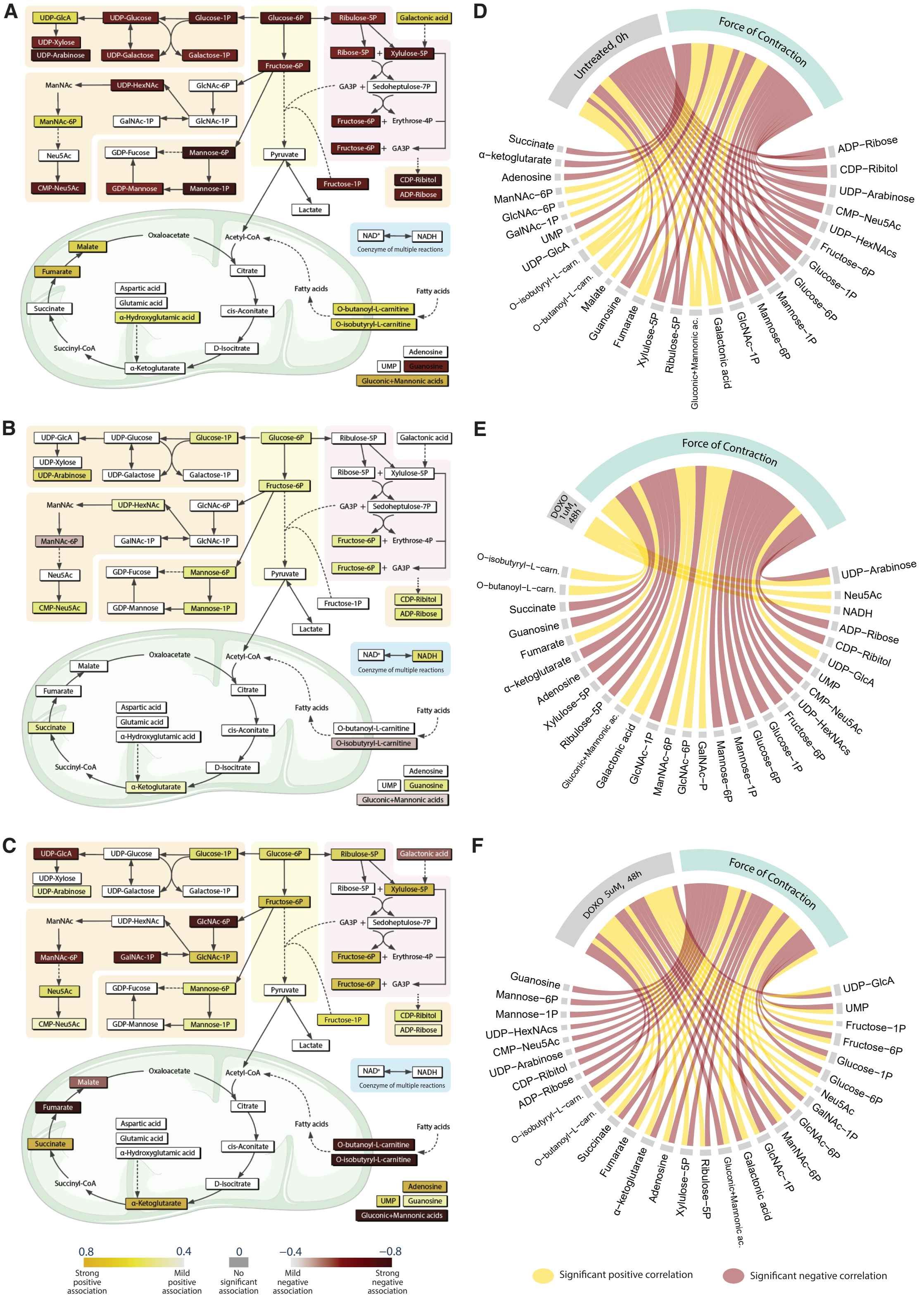

Figure 6. Contextualization of the significantly associating metabolites within cardiac cell metabolism

(A–C) Metabolic maps contextualizing the metabolites that positively (yellow) and negatively (red) associate with the condition and time point: untreated at 0 h (A), 1 μM DOXO at 48 h (B), and 5 μM DOXO at 48 h (C). The gradients of yellow (positive association) and red

(legend continued on next page)

strong positive association with untreated EHTs at 0 h and strong negative association with EHTs treated with 5 μM DOXO at 48 h. Lastly, hexosamine phosphates showed strong negative association with 5 μM DOXO treatment at 48 h, apart from GlcNAc-1P that, along with sialic acid (Neu5Ac), is instead positively associated to this dose and time.

Chord diagrams (CDs) were used to assess the effect of DOXO dose and time on metabolites strongly linked to FoC, by visually comparing (1) metabolite and *(dose,time)* relationship and (2) metabolite and FoC relationship (Figures 6D–6F). CD arm thickness represents association strength while the color denotes sign of association (yellow, positive; red, negative). When a metabolite is strongly associated with both entities of interest, namely FoC and (*dose,time*) pair, it displays two arms, which can have same color, indicating same association sign, or inversed colors, indicating a switch of association sign (SAS). A SAS indicates that a metabolite correlates with FoC while inversely correlating with treatment dose and time.

The first CD displays the results from untreated EHTs at 0 h, providing an overview of the most relevant metabolites associated with FoC. Since all these metabolites do not show a SAS, they correlate with FoC in the same way as they correlate with standard culture conditions, as expected. Once we started perturbing the system using DOXO treatments, SAS started appearing in the CDs. Specifically, in the CD of tissues treated with 1 μM DOXO for 48 h, we observed only one SAS for UDP-arabinose. NADH and Neu5Ac show strong association with treatment dose and time, however they are not directly associated with FoC (although this does not exclude secondary connection to FoC via indirect mechanisms). Within the same time frame, the EHTs treated with 5 μM DOXO manifested one order of magnitude increase in the number of metabolites displaying SAS (Figure 6F). In detail, hexose phosphates and most hexosamine phosphates show positive association with dose and time but negative association with FoC. Conversely, TCA cycle metabolites, carnitines, aldonic acid sugars, UDP-glucuronic acid, and GlcNAc-1P show negative correlation with dose and time but positive with FoC.

In conclusion, out of 48 detected metabolites we discovered 20 metabolites strongly associated with FoC and are detrimentally affected by DOXO dose and exposure time. Specifically, one metabolite displaying SAS was identified from EHTs treated with 1 μM DOXO for 48 h (Figure 6E), and 19 from EHTs treated with 5 μM for 48 h (Figure 5F).

DISCUSSION

The development of drugs is significantly hindered by the risk of undesired toxic effects, such as drug-induced cardiotoxicity, which are driven by complex molecular mechanisms often inefficiently understood. Functional 3D heart models offer new opportunities for preclinical physiological assessment of drug cardiotoxicity, but deep molecular characterization and integration of molecular and functional information remain mostly unexplored in these models. Here, we present the first integrated platform and mathematical framework for sequential and integrated assessment of functional and metabolic effects of drug cardiotoxicity in 3D-EHTs. At the core of our data integration approach is TDA, an innovative mathematical framework that identifies associations across modalities, such as FoC and metabolic profiles, thereby expediting hypothesis generation and confirmation of metabolic mechanisms linked to drug response *in vitro*.

To test our platform, we investigated DiC, a multifactorial process driven by pathophysiological mechanisms that remain incompletely understood (Mordente et al., 2009). Through secondary metabolism, DOXO can be converted into three different derivatives, previously reported in cardiac tissue: doxorubicinol (doxol), semiquinone radical, and dox-aglycones (hydroxyaglycone and deoxyaglycone) (Choksey and Timm, 2021; Clementi et al., 2003; Licata et al., 2000; Mordente et al., 2009). These derivatives, via different mechanisms, initiate cascades of reactions that lead to the production of reactive species of oxygen and nitrogen and to metabolic disturbances of several metabolic pathways, proposed as key players in DiC (Aldieri et al., 2002; Fogli et al., 2004; Mihm et al., 2002;

(negative association) colors correspond to the gradients used in the filtered HC plot (Figure 5D). Abbreviations: -1/4/6P, -1/4/6 phosphate; ADP, adenosine 5′-diphosphate; CDP, cytidine-5′-diphosphate; CMP, cytidine-5′-monophosphate; GA3P, glyceraldehyde 3-phosphate; NAD^+/NADH, oxidized/reduced forms of nicotinamide adenine dinucleotide; Neu5Ac, *N*-acetylneuraminic acid (or sialic acid); UMP, uridine 5′-monophosphate; UDP, uridine 5′-diphosphate. Background colors: green, tricarboxylic acid cycle in the mitochondrion; orange, nucleotide sugar synthesis; yellow, glycolysis; pink; pentose phosphate pathway; blue, NAD^+/NADH conversion (involved as cofactor in several reactions representing in the map).

(D–F) Chord diagrams (CDs) used to investigate the effect of dose and time on metabolites that are strongly associated with FoC. The CDs visually compare the strong correlations (positive, in yellow, or negative, in red) between metabolites and (*dose,time*) pair, with the correlation between those metabolites and FoC. Threshold of abs(corr) > 60% was applied. (D) CD built on metabolomics and functional data from untreated 3D-EHTs at 0 h. (E) CD built on data from 3D-EHTs treated with 1 μM DOXO for 48 h. (F) CD built on data from 3D-EHTs treated with 5 μM DOXO for 48 h.

Weinstein et al., 2000). Due to the targeted nature of our metabolomics methods, which covered metabolites selected *a priori* (File S1), our data-driven results corroborate some of the mechanisms previously reported to be associated to DiC in animal studies. For instance, directly or through oxidative stress, DOXO derivatives alter iron homeostasis, lipid peroxidation, and cellular energetics via mitochondrial dysfunction and inhibition of fatty acid (FA) oxidation, while also enhancing glucose uptake as compensatory mechanism for the compromised aerobic ATP production (Bauckneht et al., 2017; Govender et al., 2014; Medeiros-Lima et al., 2019; Mordente et al., 2009). The increase of glucose uptake, mostly attributed to the action of doxol, correlates with the strong association between hexose phosphates and 5 μM DOXO at 48 h, as emerged from our data-driven approach. The strong association we observed between the abundance of pentose phosphates and 5 μM DOXO treatment, which is inversely associated with FoC, aligns with previous studies reporting that DOXO-derived semiquinone radical causes lipid peroxidation, a process particularly damaging for the cardiac tissue due to its weak antioxidative defense (Chen et al., 1994; Choksey and Timm, 2021; D'Oria et al., 2020; Doroshow et al., 1980; Mordente et al., 2009; Tan et al., 2023). The increase of lipid peroxidation in turn contributes to further increase of glucose uptake, as glutathione oxidation inevitably accelerates glucose flux toward the pentose phosphate pathway (PPP) (Doroshow et al., 1980; Mordente et al., 2009).

Additionally, mitochondrial aerobic metabolism is known to be heavily impacted by DOXO (Choksey and Timm, 2021; Mordente et al., 2009), and our findings are in line with this notion. For example, our analysis highlighted NADH as strongly associated with 1 μM DOXO dose at 48 h, but not with 5 μM. This observation is consistent with a previously proposed hypothesis (Marcillat et al., 1989), whereby low concentrations of DOXO cause site-specific oxidative damage to the NADH oxidation pathway, while higher concentrations affect other respiratory chain complexes without the involvement of DOXO redox cycling. Our results on succinate and its downstream metabolites, fumarate and malate, also align with this hypothesis. In our analysis, a strong accumulation of succinate is detected when EHTs are treated with 5 μM DOXO at 48 h, accompanied by reduced levels of fumarate and malate, which may indirectly point toward impaired mitochondrial functionality under these conditions. This also aligns with a recently published study (Schwach et al., 2024), reporting mitochondrial damage in 3D-EHTs following treatment with 5 μM DOXO for 24 h, including reduction of respiration via complex I/II and oxidative phosphorylation capacity. Regarding FA oxidation, although our targeted MS methods only included two carnitine esters involved in the transport of FA into the mitochondria, we observed a mild negative effect at 1 μM DOXO and a strong negative effect at 5 μM DOXO at 48 h. These findings are compatible with previous reports suggesting that DOXO compromises FA homeostasis and utilization in cardiac cells (Carvalho et al., 2010; Hong et al., 2002; Strigun et al., 2012). While dedicated studies specifically targeting mitochondrial metabolism and FA oxidation are necessary to further validate our findings, our data-driven approach has identified multiple metabolic responses with distinct time- and dose-specific dynamics, consistently with current hypotheses on DiC pathophysiology.

Further, our approach also enabled the identification of metabolic targets in pathways previously unexplored in the context of DiC. For instance, UDP-glucuronic acid stood out among the other nucleotides. Although direct glucuronidation of DOXO has been ruled out based on its chemical composition (Erttmann et al., 1988; Krarup-Hansen et al., 1988; Weenen et al., 1984), we detected a strong positive correlation with FoC and strong negative correlation with DOXO dose and exposure time (Figures 6A–6C, File S3), suggesting that changes in the level of this metabolite are linked to FoC and to the treatment, warranting further investigations. Likewise, some compounds of the hexosamine biosynthesis pathway (HBP), such as GlcNAc-1-phosphate and Neu5Ac, also showed strong association with DOXO treatment. Interestingly, to our knowledge, the compounds have not yet been explored as metabolic targets of DiC. As several studies reported alterations in gene expression caused by DOXO and its derivatives (Ito et al., 1990; Matthews et al., 2024; McSweeney et al., 2019; Stamm et al., 2021; Tokarska-Schlattner et al., 2010), investigating the disturbance of HBP, which directly links metabolic state and gene expression via O-GlcNAcylation, should be explored in more detail as a potential underlying mechanism of gene expression alteration in DiC. Lastly, aldonic acid sugars were also found significantly associated with FoC and oppositely associated with both DOXO doses, particularly with 5 μM. This could be linked to the enhanced flux of glucose toward glycolysis and PPP to compensate for defective ATP production.

In conclusion, our study sets the stage for detailed molecular and functional investigation of drug effects and cardiotoxicity in 3D cardiac models, bringing preclinical assessment one step closer to effective prediction of clinical relevance. Through our approach, we were able to explore the complex metabolic modulation of the CCM triggered by DOXO treatment and to identify 20 metabolites strongly related with FoC and altered by DOXO dose and exposure time. With the rise of metabolic modulation as a new strategy to counteract DOXO toxicity (Diamanti et al., 2014; Djabir et al., 2017; Ekinci Akdemir et al., 2021; Hosseini et al., 2022; Mandziuk et al., 2015;

Mantawy et al., 2017; Warpe et al., 2015; Zhang et al., 2017; Zhu et al., 2017), we speculate that these metabolites could hold potential as target for metabolic DiC modulation and should be further investigated.

Beyond DiC, this study showcased how TDA successfully revealed both dose- and time-dependent modulation of the CCM, and how node enrichment can transform crucial categorical information into quantitative data that can then be incorporated in downstream data analysis to identify relevant molecular targets. While the current study presents a proof of concept dataflow for hypothesis generation from small datasets with varying modalities, our versatile workflow could be expanded and adapted to accommodate larger omics datasets. In fact, data size is not a limiting factor for successful application of the proposed TDA-based workflow in larger preclinical and clinical studies. Furthermore, our workflow could aid the holistic exploration of data derived from other cutting edge omics technologies, such as proteomics and lipidomics, while enabling integration with functional readouts that are the key characteristics of 3D-EHTs. The choice of testing our analytical framework in 3D-EHTs stemmed from several advantages. First, the cellular composition in 3D-EHTs is controlled, expediting the discovery of mechanistic insights in CMs as the metabolomics approach deployed here cannot distinguish the contributions of different cell types. Second, 3D-EHT generation is standardized, ensuring metabolic comparability across replicates. Third, we previously established a non-destructive FoC recording method, enabling functional measurements immediately before metabolite extraction. Importantly, our TDA-guided analytical framework is also suitable for the integration of functional and omics data from other types of cardiac models, such as cardiospheres and cardiac organoids, which are increasingly used to investigate drug effects and toxicity (Chen et al., 2023; Hoang et al., 2021; Richards et al., 2020).

This study offers the first proof of concept of how the combination of advanced cardiac tissue engineering, state-of-the-art omics technologies and novel mathematical approaches can deepen and expedite preclinical investigations of drug effects and cardiotoxicity, offering new strategies to enhance the translational power and to reduce the failure rate of preclinical drug development.

METHODS

Cardiomyogenic differentiation and 3D-EHT generation

The commercial hiPSC line "WTC" (GM25256, Coriell Institute) was used to generate CMs, as previously described (Birket et al., 2015; Rivera-Arbeláez et al., 2022). In brief, after 13-day culture in BSA polyvinylalcohol essential lipids medium (BPEL) medium, beating CMs underwent metabolic purification based on CM-specific medium (CM medium) without glucose but supplemented with 5 mM sodium DL-lactate solution (60%, Sigma-Aldrich) until day 17 (Figure 2A). Then, CMs recovered in CM medium with 4.5 mM glucose for three days (day 20) until cryopreservation. To estimate batch purity before freezing, human cTnT expression was evaluated using a human anti-cTnT Antibody-VioBlue (excitation 400nm, emission 450, Miltenyi Biotec) through a MACSQuant VYB flow cytometer (filter set 450/50nm, Miltenyi Biotec), and the plots were analyzed with FlowLogic software. Only batches with more than 90% cTnT-positive CMs were used for downstream applications. 3D-EHTs were generated as previously described (Ribeiro et al., 2022), with a variation in the cell-matrix mix composition. First, the extracellular matrix (ECM) mixture was prepared by combining 2X CM medium, 10% fibrinogen (final concentration 2 mg/mL, Sigma-Aldrich), 10% Matrigel (final concentration 1 mg/mL, Corning) and 1% aprotinin (final concentration 2.5 μg/mL, Sigma). Next, the ECM mixture was combined with CMs and human adult CFs (line C-12375, Promocell) with ratio 100:3. Just before seeding the cell mixture in the gelatin slots, 0.3% thrombin (Sigma) was added to enable the jellification of the mixture around the pillars' heads. In this study, the PDMS block was customized to accommodate only one EHT per holder/well, to enable FoC measurement and metabolite extraction from each individual EHT. After formation, the EHTs were maintained at 37°C and 5% CO_2 and the medium was refreshed after 24 h. Then, the medium was refreshed every 2 days until day 10 after tissue casting (corresponding to day 30 in Figure 1A).

DOXO treatment

On day 11 after tissue formation (corresponding to day 31 in Figure 1A), three paced, untreated EHTs ($n = 3$) immediately underwent metabolic extraction after FoC recording, representing time point 0 (baseline). The remainig EHTs were divided into three groups and cultured in CM medium supplemented with: (a) 7.05 mM DMSO (1:2000 dilution) as control condition, (b) 1 μM, or (c) 5 μM DOXO. For the second time point (24 h), nine EHTs ($n = 3$/condition) were used to record contraction force and for sequential extraction. On the same day, the nine EHTs ($n = 3$/condition) meant for the third time point were refreshed with the corresponding medium and cultured for an additional 24 h. At the third time point (48 h) the last 9 EHTs were used to record contraction force and sequential metabolite extraction.

FoC acquisition

The contractile activity of each 3D-EHT was measured upon electrical pacing using a Nikon Ti2-E inverted microscope with a high-speed camera Prime BSI (Photometrics)

at 100 fps with 2× magnification. Electrical pacing was performed using two platinum electrodes (Advent Research Materials) connected to a custom-made pacing device at 2 Hz (10 ms biphasic pulses, 4–5 V/cm) for 10 s. EHTs were kept at 37C and 5% CO_2 during recording. The videos were then uploaded to the *EHT Analysis* software (Rivera-Arbeláez et al., 2022), which converted the spatial displacement of the pillars' heads into FoC.

Videos were recorded at time point 0 for the untreated tissues (*n=3,* which underwent metabolic extraction immediately after) and the tissues meant to undergo treatments ($n = 18$), to establish a baseline. At time point 24 h, the videos for the DMSO treated EHTs (control, $n = 3$) and DOXO treated EHTs ($n=6$) were recorded just before metabolic extraction. The same steps were performed at time point 48 h on the remaining DMSO treated ($n = 3$) and DOXO treated EHTs ($n = 6$).

On-pillar extraction of polar metabolites

To extract polar metabolites while preserving tissue integrity and minimizing handling, which increases variability, we optimized an on-pillar metabolite extraction. After electrical pacing and video recording for FoC quantification, the EHT (still mounted on the pillars) was transferred to a new well from a 12-well plate filled with 4 mL of washing buffer (75 mM ammonium carbonate, with pH adjusted to 7.2–7.4 with AA), ensuring that the tissue was fully submerged in the buffer for about 5 s (Figure 1). Next, the EHT (still on the pillars) was transferred to a second well filled with 4 mL of washing buffer for the second wash. Lastly, the EHT was transferred into a well containing 1.7 mL of −20C cold extraction buffer (40:40:20 v/v acetonitrile:methanol:ultra-pure water), while the plate was kept on ice during incubation. To identify the minimal incubation time yielding maximal extraction of polar metabolites, seven different incubation times (5, 7.5, 10, 12.5, 15, 17.5, and 20 min) were tested prior to performing the treatment experiment (see results).

After incubation, the EHT still mounted on pillars (Figure S1) was removed from the extraction buffer, detached from the pillars using tweezers and snap-frozen in liquid nitrogen for later dsDNA quantification. The extraction buffer (containing the extracted metabolites) was centrifuged at 14,000 rpm for 5 min, and the resulting supernatant was transferred to a new tube and concentrated overnight *in vacuo* using an SC100 SpeedVac Concentrator equipped with a refrigerated condensation trap (Savant) (Figure 1A). After concentration, the samples were stored at −80C until MS analysis.

Post-extraction dsDNA quantification

To quantify the double strand DNA (dsDNA) from the leftover tissues after metabolic extraction (Figure S1), the tissues were thawed on ice and grinded in 25 μL of 8M urea using a microtube-fitting plastic pestle (Cytiva) until the tissues were fully homogenized. Next, 2 μL of each homogenate were used to quantify the dsDNA content via a NanoDrop spectrophotometer (Thermo Fisher Scientific) at 260 nm absorbance. Likewise, the dsDNA content in the metabolic extract was also measured. The dsDNA measurements from the tissue homogenate and from the metabolic extract of the same EHT were summed to determine the total dsDNA content, which was then used to normalize MS data (File S1, Figure 3A).

Metabolomics profiling

TEA-based and TBA-based LC-MS/MS analyses

The metabolite extracts were dissolved in 100 μL of Milli-Q water prior to MS analysis (Figure 1B).

For the TEA-based method, 10 μL of extract was loaded onto an Agilent 1290 ultra-high-performance liquid chromatography (UHPLC) column connected to an Agilent 6490A tandem quadrupole mass spectrometer (MS/MS), operated in dynamic multiple-reaction monitoring (MRM) mode, as previously reported (Conte et al., 2023a, 2023b).

For the TBA-based method, 20 μL of extracts were injected into the same UHPLC-MS/MS system, also operated in MRM, using the acquisition method previously reported (van Scherpenzeel et al., 2022).

The chromatography settings, buffer composition and list of transitions for both methods are reported in File S1.

AA-based LC-MS/MS analysis

The metabolic extract (20 μL) was transferred to a 96-well plate and diluted with 20 μL of stable isotope solution, containing three internal standards: 3 μM U-$^{13}C_6$-citrate (Toronto Research Chemicals), 6 μM U-$^{13}C_5$ 2-ketoglutarate, and 3 μM of U-$^{13}C_4$ succinate (Cambridge Isotope Laboratories). To mix the diluted samples, the plate was centrifuged for 3 min at 2,000 rpm at 15C, and shaken for 3 min at 100 rpm. Then, 1 μL of each sample was injected in a Waters I-Class Acquity chromatographic system, fitted with a peek-lined Inert Sustain AQ-c18 (2.1 × 100 mm dp 3 μm) column and connected to a Waters Xevo TQSμ mass spectrometer, and analyzed using the method previously described (Vriend et al., 2019). The buffer composition, gradient and list of transitions are reported in File S1.

Metabolomics data processing

The chromatograms from the three LC-MS/MS methods were integrated via Skyline (v.20.2) using the transition lists of the target compounds (File S1). The resulting peak areas were normalized on dsDNA content. For each metabolite, *Z* scores of normalized peak values were calculated (see boxplots in Figures S4–S6) using "*Z* score" module in "SciPy" library (v.1.10.1) and arranged as column vectors with a shared row vector space, which in our case

corresponded to sample replicates at different doses and time. Each metabolomics dataset was considered as a point cloud sampled from a high-dimensional manifold, embedded in a topological space with a separate dimension for each metabolite.

Here, $\underline{X}$ denotes the set of Z scores as:

$$\underline{X} = \{X^i \in \mathbb{R}^{D_i} \text{ for } 1 \leq i \leq 3\} \qquad \text{(Equation 1)}$$

where D_i is the number of metabolites in the i-th dataset.

Data integration via TDA

To recover the topology of the shared space across datasets, we employed the *Mapper* algorithm (Singh et al., 2007). The typical TDA workflow starts with selecting a reference metric space equipped with a measure of proximity or similarity between samples in the low-dimensional space (Figure 4A). We selected UMAP as a dimensionality-reduction approach due to its ability to preserve the samples proximity present in the original high-dimensional manifold (McInnes et al., 2018).

Here, $\underline{F}$ denotes the set of low-dimensional embeddings of three metabolomics datasets as:

$$\underline{F} = \{f_i : X^i \in \mathbb{R}^{D_i} \rightarrow Y^i \in \mathbb{R}^2 \text{ for } 1 \leq i \leq 3\} \qquad \text{(Equation 2)}$$

with f_i indicating a function, commonly referred as filter function (or topological lens/reference map) in TDA, that projects the original high-dimensional space X^i to two-dimensional *filtered* space Y^i. The resulting UMAP projections are shown in Figures 3E–3G. To facilitate downstream analysis, various layers of information, namely the topological space $\underline{X}$, its map $\underline{F}$ and the sample metadata, were further collated as annotated data objects (Virshup et al., 2021).

Next, with UMAP projections generated as per Equation 2, a composite filtered space was constructed via horizontal concatenation:

$$\underline{\boldsymbol{Y}} = [Y^1 | Y^2 | Y^3] \in \mathbb{R}^6 \qquad \text{(Equation 3)}$$

with $Y^i, 1 \leq i \leq 3$ sharing their row vector space (samples).

We used $\{U_k^l\}_{1 \leq k \leq K}$ to indicate K sequential overlapping intervals covering the range of values in each column l of $\underline{\boldsymbol{Y}}$, and $\underline{U} \in \mathbb{R}^6$ to denote a multi-dimensional cover of $\underline{\boldsymbol{Y}}$ constructed as a set of overlapping hypercubes:

$$\underline{U} = \{(U_k^1 \times \cdots \times U_k^l \times \cdots \times U_k^6) \text{ for } 1 \leq k \leq K\} \qquad \text{(Equation 4)}$$

The *Mapper* algorithm was then used to convert the cover $\underline{U}$ into a simplicial complex or nerve $N(\underline{U}, \underline{F})$ that encodes the structure of $\underline{F}$ by pulling back the cover of the space $\underline{Y}$ to a cover on $\underline{X}$ through $\underline{F}$, followed by sample clustering and computing a neighborhood graph. The cover $\underline{U}$ was subdivided into three hypercubes with 30% overlap. Module "CubicalCover" in *giotto-tda package* (Tauzin et al., 2021) was used to perform the data integration and to construct the TCN with sample connectivity (File S3).

Correlation-based inference framework to detect associations between metabolites and DOXO dose and time

Following the construction of the TCN, sub-sets of samples clustered in each node were extracted. In the TCN, sample clusters associated with connected nodes share one or more samples. For each sample cluster, the mean values for the corresponding original metabolomics data were calculated and further referred to as node values (or node coordinates) in the high-dimensional metabolic space. For categorical metadata (such as treatment type, time point or combination of both), the percentage of samples with associated parameter value in each cluster served as a node value. Next, a new data matrix with node values across the entire topological network was constructed (File S4). To estimate the associations/relationships between pairs of data and metadata, Spearman's rank correlation matrix (Dodge, 2008), based on node values, was calculated and benchmarked as described in Text S1.

To identify groups of features exhibiting similar patterns of relationships with treatment dose and time, a HC of the corresponding Spearman's rank correlations based on node enrichment was performed (Figure S3). Then, to identify metabolites significantly associated with treatment and time, a three-step filtration was applied: (1) metabolites with $p < 0.05$ in at least one condition were retained; (2) from these, only those with an absolute correlation > 60% were kept; and (3) correlation values for metabolites not meeting these criteria were set to zero (Figure 5D).

Lastly, to visualize statistically significant inferences, positive or negative, we generated CDs displaying the associations between metabolites, FoC and treatment *(dose,time)* pairs. Only correlations with absolute values above 60% were displayed. CDs were built with "chordDiagram" function in *R package "circlize" (v.0.4.15).*

RESOURCE AVAILABILITY

Lead contact

Requests for further information and resources should be directed to and will be fulfilled by the lead contact, Prof. Robert Passier (robert.passier@utwente.nl).

Materials availability

This study did not generate new unique reagents.

Data and code availability

Raw data files have been deposited in the FAIR-compliant repository MetaboLights, under the accession number MetaboLights: MTBLS13225, and are publicly available as of the date of publication. The supplemental files (1–4) and the original code have been deposited at FigShare.com, under the DOI https://doi.org/10.6084/m9.figshare.27985364.v2, and are publicly available at as of the date of publication.

ACKNOWLEDGMENTS

This study was financially supported by the Twente University & RadBoudumc Opportunities (TURBO) grant 2020-21 (to R.P., D.J.L., and F.C.), Prinses Beatrix Spierfonds ("Op weg naar therapie" grant W.OR22-14 to F.C.), the ZonMw (VICI grant 09150182010010 to D.J.L.), the European Research Council ("Heart2Beat" grant no. 101098372 to R.P.), and Metakids and United for Metabolic Diseases (research grant to D.J.L. and D.K.S).

Figures 1B, 2A, 3A, 4A, and the graphical abstract were generated using BioRender (BioRender.com).

AUTHOR CONTRIBUTIONS

Conceptualization: F.C., D.K.S., D.J.L., and R.P. Methodology: F.C., D.K.S., C.C.-F., D.S., and A.v.R. Investigation: F.C. and D.K.S. Visualization: F.C. and D.K.S. Supervision: D.J.L. and R.P. Writing – original draft: F.C., D.K.S., D.J.L., and R.P. Writing – review and editing: F.C., D.K.S., C.C.-F., D.S., A.v.R., D.J.L., and R.P.

The corresponding authors, R.P. (senior expert in cardiac models) and D.J.L. (senior expert in MS-based metabolomics), have equally contributed to the supervision of the experimental work, the assurance of data integrity and storage, and the preparation of the manuscript.

DECLARATION OF INTERESTS

R.P. is a cofounder of Pluriomics (Ncardia) and River BioMedics BV. D.K.S. is a cofounder of Multicore Dynamics Ltd.

SUPPLEMENTAL INFORMATION

Supplemental information can be found online at https://doi.org/10.1016/j.stemcr.2025.102725.

Received: April 11, 2025
Revised: November 4, 2025
Accepted: November 5, 2025
Published: December 4, 2025

REFERENCES

Ahuja, P., Perriard, E., Perriard, J.C., and Ehler, E. (2004). Sequential myofibrillar breakdown accompanies mitotic division of mammalian cardiomyocytes. J. Cell Sci. *117*, 3295–3306. https://doi.org/10.1242/jcs.01159.

Aldieri, E., Bergandi, L., Riganti, C., Costamagna, C., Bosia, A., and Ghigo, D. (2002). Doxorubicin induces an increase of nitric oxide synthesis in rat cardiac cells that is inhibited by iron supplementation. Toxicol. Appl. Pharmacol. *185*, 85–90. https://doi.org/10.1006/taap.2002.9527.

Amézquita, E.J., Quigley, M.Y., Ophelders, T., Munch, E., and Chitwood, D.H. (2020). The shape of things to come: Topological data analysis and biology, from molecules to organisms. Dev. Dyn. *249*, 816–833. https://doi.org/10.1002/dvdy.175.

Avagimyan, A., Kakturskiy, L., Heshmat-Ghahdarijani, K., Pogosova, N., and Sarrafzadegan, N. (2022). Anthracycline Associated Disturbances of Cardiovascular Homeostasis. Curr. Probl. Cardiol. *47*, 100909. https://doi.org/10.1016/j.cpcardiol.2021.100909.

Barpe, D.R., Rosa, D.D., and Froehlich, P.E. (2010). Pharmacokinetic evaluation of doxorubicin plasma levels in normal and overweight patients with breast cancer and simulation of dose adjustment by different indexes of body mass. Eur. J. Pharm. Sci. *41*, 458–463. https://doi.org/10.1016/j.ejps.2010.07.015.

Bauckneht, M., Ferrarazzo, G., Fiz, F., Morbelli, S., Sarocchi, M., Pastorino, F., Ghidella, A., Pomposelli, E., Miglino, M., Ameri, P., et al. (2017). Doxorubicin Effect on Myocardial Metabolism as a Prerequisite for Subsequent Development of Cardiac Toxicity: A Translational (18)F-FDG PET/CT Observation. J. Nucl. Med. *58*, 1638–1645. https://doi.org/10.2967/jnumed.117.191122.

Birket, M.J., Ribeiro, M.C., Kosmidis, G., Ward, D., Leitoguinho, A.R., van de Pol, V., Dambrot, C., Devalla, H.D., Davis, R.P., Mastroberardino, P.G., et al. (2015). Contractile Defect Caused by Mutation in MYBPC3 Revealed under Conditions Optimized for Human PSC-Cardiomyocyte Function. Cell Rep. *13*, 733–745. https://doi.org/10.1016/j.celrep.2015.09.025.

Bordoni, A., Biagi, P., and Hrelia, S. (1999). The impairment of essential fatty acid metabolism as a key factor in doxorubicin-induced damage in cultured rat cardiomyocytes. Biochim. Biophys. Acta *1440*, 100–106. https://doi.org/10.1016/s1388-1981(99)00113-4.

Bugger, H., Guzman, C., Zechner, C., Palmeri, M., Russell, K.S., and Russell, R.R., 3rd. (2011). Uncoupling protein downregulation in doxorubicin-induced heart failure improves mitochondrial coupling but increases reactive oxygen species generation. Cancer Chemother. Pharmacol. *67*, 1381–1388. https://doi.org/10.1007/s00280-010-1441-7.

Carrière, M., Michel, B., and Oudot, S. (2018). Statistical analysis and parameter selection for mapper. J. Mach. Learn. Res. *19*, 478–516.

Carvalho, R.A., Sousa, R.P.B., Cadete, V.J.J., Lopaschuk, G.D., Palmeira, C.M.M., Bjork, J.A., and Wallace, K.B. (2010). Metabolic remodeling associated with subchronic doxorubicin cardiomyopathy. Toxicology *270*, 92–98. https://doi.org/10.1016/j.tox.2010.01.019.

Chazal, F., and Michel, B. (2021). An Introduction to Topological Data Analysis: Fundamental and Practical Aspects for Data Scientists. Front. Artif. Intell. *4*, 667963. https://doi.org/10.3389/frai.2021.667963.

Chen, X., Lu, N., Huang, S., Zhang, Y., Liu, Z., and Wang, X. (2023). Assessment of doxorubicin toxicity using human cardiac organoids: A novel model for evaluating drug cardiotoxicity. Chem. Biol. Interact. *386*, 110777. https://doi.org/10.1016/j.cbi.2023.110777.

Chen, Y., Saari, J.T., and Kang, Y.J. (1994). Weak antioxidant defenses make the heart a target for damage in copper-deficient rats. Free Radic. Biol. Med. *17*, 529–536. https://doi.org/10.1016/0891-5849(94)90092-2.

Choksey, A., and Timm, K.N. (2021). Cancer Therapy-Induced Cardiotoxicity-A Metabolic Perspective on Pathogenesis, Diagnosis and Therapy. Int. J. Mol. Sci. *23*, 441. https://doi.org/10.3390/ijms23010441.

Clementi, M.E., Giardina, B., Di Stasio, E., Mordente, A., and Misiti, F. (2003). Doxorubicin-derived metabolites induce release of cytochrome C and inhibition of respiration on cardiac isolated mitochondria. Anticancer Res. *23*, 2445–2450.

Conte, F., Ashikov, A., Mijdam, R., van de Ven, E.G.P., van Scherpenzeel, M., Veizaj, R., Mahalleh-Yousefi, S.P., Post, M.A., Huijben, K., Panneman, D.M., et al. (2023). In Vitro Skeletal Muscle Model of PGM1 Deficiency Reveals Altered Energy Homeostasis. Int. J. Mol. Sci. *24*, 8247. https://doi.org/10.3390/ijms24098247.

Conte, F., Noga, M.J., van Scherpenzeel, M., Veizaj, R., Scharn, R., Sam, J.E., Palumbo, C., van den Brandt, F.C.A., Freund, C., Soares, E., et al. (2023). Isotopic Tracing of Nucleotide Sugar Metabolism in Human Pluripotent Stem Cells. Cells *12*, 1765. https://doi.org/10.3390/cells12131765.

D'Oria, R., Schipani, R., Leonardini, A., Natalicchio, A., Perrini, S., Cignarelli, A., Laviola, L., and Giorgino, F. (2020). The Role of Oxidative Stress in Cardiac Disease: From Physiological Response to Injury Factor. Oxid. Med. Cell. Longev. *2020*, 5732956. https://doi.org/10.1155/2020/5732956.

Diamanti, J., Mezzetti, B., Giampieri, F., Alvarez-Suarez, J.M., Quiles, J.L., Gonzalez-Alonso, A., Ramirez-Tortosa, M.d.C., Granados-Principal, S., Gonzáles-Paramás, A.M., Santos-Buelga, C., and Battino, M. (2014). Doxorubicin-induced oxidative stress in rats is efficiently counteracted by dietary anthocyanin differently enriched strawberry (Fragaria × ananassa Duch.). J. Agric. Food Chem. *62*, 3935–3943. https://doi.org/10.1021/jf405721d.

Dirnagl, U., Duda, G.N., Grainger, D.W., Reinke, P., and Roubenoff, R. (2022). Reproducibility, relevance and reliability as barriers to efficient and credible biomedical technology translation. Adv. Drug Deliv. Rev. *182*, 114118. https://doi.org/10.1016/j.addr.2022.114118.

Djabir, Y.Y., Arsyad, M.A., Sartini, S., and Lallo, S. (2017). Potential Roles of Kleinhovia hospita L. Leaf Extract in Reducing Doxorubicin Acute Hepatic, Cardiac and Renal Toxicities in Rats. Pharmacogn. Res. *9*, 168–173. https://doi.org/10.4103/pr.pr_129_16.

Doroshow, J.H., Locker, G.Y., and Myers, C.E. (1980). Enzymatic defenses of the mouse heart against reactive oxygen metabolites: alterations produced by doxorubicin. J. Clin. Investig. *65*, 128–135. https://doi.org/10.1172/jci109642.

Ebbels, T.M.D., van der Hooft, J.J.J., Chatelaine, H., Broeckling, C., Zamboni, N., Hassoun, S., and Mathé, E.A. (2023). Recent advances in mass spectrometry-based computational metabolomics. Curr. Opin. Chem. Biol. *74*, 102288. https://doi.org/10.1016/j.cbpa.2023.102288.

Ekinci Akdemir, F.N., Yildirim, S., Kandemir, F.M., Tanyeli, A., Küçükler, S., and Bahaeddin Dortbudak, M. (2021). Protective effects of gallic acid on doxorubicin-induced cardiotoxicity; an experimental study. Arch. Physiol. Biochem. *127*, 258–265. https://doi.org/10.1080/13813455.2019.1630652.

Erttmann, R., Erb, N., Steinhoff, A., and Landbeck, G. (1988). Pharmacokinetics of doxorubicin in man: dose and schedule dependence. J. Cancer Res. Clin. Oncol. *114*, 509–513. https://doi.org/10.1007/bf00391502.

Fogli, S., Nieri, P., and Breschi, M.C. (2004). The role of nitric oxide in anthracycline toxicity and prospects for pharmacologic prevention of cardiac damage. FASEB J. *18*, 664–675. https://doi.org/10.1096/fj.03-0724rev.

Govender, J., Loos, B., Marais, E., and Engelbrecht, A.M. (2014). Mitochondrial catastrophe during doxorubicin-induced cardiotoxicity: a review of the protective role of melatonin. J. Pineal Res. *57*, 367–380. https://doi.org/10.1111/jpi.12176.

Greek, R., and Menache, A. (2013). Systematic reviews of animal models: methodology versus epistemology. Int. J. Med. Sci. *10*, 206–221. https://doi.org/10.7150/ijms.5529.

Guo, Y., and Pu, W.T. (2020). Cardiomyocyte Maturation: New Phase in Development. Circ. Res. *126*, 1086–1106. https://doi.org/10.1161/circresaha.119.315862.

Henriksen, P.A. (2018). Anthracycline cardiotoxicity: an update on mechanisms, monitoring and prevention. Heart *104*, 971–977. https://doi.org/10.1136/heartjnl-2017-312103.

Hoang, P., Kowalczewski, A., Sun, S., Winston, T.S., Archilla, A.M., Lemus, S.M., Ercan-Sencicek, A.G., Gupta, A.R., Liu, W., Kontaridis, M.I., et al. (2021). Engineering spatial-organized cardiac organoids for developmental toxicity testing. Stem Cell Rep. *16*, 1228–1244. https://doi.org/10.1016/j.stemcr.2021.03.013.

Hoekstra, R., Kiers, H., and Johnson, A. (2012). Are assumptions of well-known statistical techniques checked, and why (not)? Front. Psychol. *3*, 137. https://doi.org/10.3389/fpsyg.2012.00137.

Hong, Y.M., Kim, H.S., and Yoon, H.R. (2002). Serum lipid and fatty acid profiles in adriamycin-treated rats after administration of L-carnitine. Pediatr. Res. *51*, 249–255. https://doi.org/10.1203/00006450-200202000-00020.

Hossain, S. (2019). Visualization of Bioinformatics Data with Dash Bio. In scipy. https://doi.org/10.25080/Majora-7ddc1dd1-012.

Hosseini, A., Safari, M.K., Rajabian, A., Boroumand-Noughabi, S., Eid, A.H., Al Dhaheri, Y., Gumpricht, E., and Sahebkar, A. (2022). Cardioprotective Effect of Rheum turkestanicum Against Doxorubicin-Induced Toxicity in Rats. Front. Pharmacol. *13*, 909079. https://doi.org/10.3389/fphar.2022.909079.

Ito, H., Miller, S.C., Billingham, M.E., Akimoto, H., Torti, S.V., Wade, R., Gahlmann, R., Lyons, G., Kedes, L., and Torti, F.M. (1990). Doxorubicin selectively inhibits muscle gene expression in cardiac muscle cells in vivo and in vitro. Proc. Natl. Acad. Sci. USA *87*, 4275–4279. https://doi.org/10.1073/pnas.87.11.4275.

Joshi, M.A., and Joshi, D.L. (2020). Topological Data Analysis Based Feature Selection for Predicting Fatigue Strength of Steel Using Machine Learning. IOP Conf. Ser. Mater. Sci. Eng. *810*, 012083. https://doi.org/10.1088/1757-899X/810/1/012083.

Kaur, P., Singh, A., and Chana, I. (2021). Computational Techniques and Tools for Omics Data Analysis: State-of-the-Art, Challenges, and Future Directions. Arch. Comput. Methods Eng. *28*, 4595–4631. https://doi.org/10.1007/s11831-021-09547-0.

Krarup-Hansen, A., Wassermann, K., Rasmussen, S.N., and Dalmark, M. (1988). Pharmacokinetics of doxorubicin in man with induced acid or alkaline urine. Acta Oncol. *27*, 25–30. https://doi.org/10.3109/02841868809090314.

Licata, S., Saponiero, A., Mordente, A., and Minotti, G. (2000). Doxorubicin metabolism and toxicity in human myocardium: role of cytoplasmic deglycosidation and carbonyl reduction. Chem. Res. Toxicol. *13*, 414–420. https://doi.org/10.1021/tx000013q.

Linders, A.N., Dias, I.B., López Fernández, T., Tocchetti, C.G., Bomer, N., and Van der Meer, P. (2024). A review of the pathophysiological mechanisms of doxorubicin-induced cardiotoxicity and aging. NPJ Aging *10*, 9. https://doi.org/10.1038/s41514-024-00135-7.

López de Maturana, E., Alonso, L., Alarcón, P., Martín-Antoniano, I.A., Pineda, S., Piorno, L., Calle, M.L., and Malats, N. (2019). Challenges in the Integration of Omics and Non-Omics Data. Genes *10*, 238. https://doi.org/10.3390/genes10030238.

Lotrionte, M., Biondi-Zoccai, G., Abbate, A., Lanzetta, G., D'Ascenzo, F., Malavasi, V., Peruzzi, M., Frati, G., and Palazzoni, G. (2013). Review and meta-analysis of incidence and clinical predictors of anthracycline cardiotoxicity. Am. J. Cardiol. *112*, 1980–1984. https://doi.org/10.1016/j.amjcard.2013.08.026.

Lum, P.Y., Singh, G., Lehman, A., Ishkanov, T., Vejdemo-Johansson, M., Alagappan, M., Carlsson, J., and Carlsson, G. (2013). Extracting insights from the shape of complex data using topology. Sci. Rep. *3*, 1236. https://doi.org/10.1038/srep01236.

Mandziuk, S., Gieroba, R., Korga, A., Matysiak, W., Jodlowska-Jedrych, B., Burdan, F., Poleszak, E., Kowalczyk, M., Grzycka-Kowalczyk, L., Korobowicz, E., et al. (2015). The differential effects of green tea on dose-dependent doxorubicin toxicity. Food Nutr. Res. *59*, 29754. https://doi.org/10.3402/fnr.v59.29754.

Mantawy, E.M., Esmat, A., El-Bakly, W.M., Salah ElDin, R.A., and El-Demerdash, E. (2017). Mechanistic clues to the protective effect of chrysin against doxorubicin-induced cardiomyopathy: Plausible roles of p53, MAPK and AKT pathways. Sci. Rep. *7*, 4795. https://doi.org/10.1038/s41598-017-05005-9.

Marcillat, O., Zhang, Y., and Davies, K.J.A. (1989). Oxidative and non-oxidative mechanisms in the inactivation of cardiac mitochondrial electron transport chain components by doxorubicin. Biochem. J. *259*, 181–189. https://doi.org/10.1042/bj2590181.

Matthews, E.R., Johnson, O.D., Horn, K.J., Gutiérrez, J.A., Powell, S.R., and Ward, M.C. (2024). Anthracyclines induce cardiotoxicity through a shared gene expression response signature. PLoS Genet. *20*, e1011164. https://doi.org/10.1371/journal.pgen.1011164.

McInnes, L., Healy, J., and Melville, J. (2018). UMAP: Uniform Manifold Approximation and Projection for Dimension Reduction. Preprint at arXiv. https://doi.org/10.48550/arXiv.1802.03426.

McSweeney, K.M., Bozza, W.P., Alterovitz, W.-L., and Zhang, B. (2019). Transcriptomic profiling reveals p53 as a key regulator of doxorubicin-induced cardiotoxicity. Cell Death Discov. *5*, 102. https://doi.org/10.1038/s41420-019-0182-6.

Medeiros-Lima, D.J.M., Carvalho, J.J., Tibirica, E., Borges, J.P., and Matsuura, C. (2019). Time course of cardiomyopathy induced by doxorubicin in rats. Pharmacol. Rep. *71*, 583–590. https://doi.org/10.1016/j.pharep.2019.02.013.

Mihm, M.J., Yu, F., Weinstein, D.M., Reiser, P.J., and Bauer, J.A. (2002). Intracellular distribution of peroxynitrite during doxorubicin cardiomyopathy: evidence for selective impairment of myofibrillar creatine kinase. Br. J. Pharmacol. *135*, 581–588. https://doi.org/10.1038/sj.bjp.0704495.

Milani-Nejad, N., and Janssen, P.M.L. (2014). Small and large animal models in cardiac contraction research: advantages and disadvantages. Pharmacol. Ther. *141*, 235–249. https://doi.org/10.1016/j.pharmthera.2013.10.007.

Mitry, M.A., and Edwards, J.G. (2016). Doxorubicin induced heart failure: Phenotype and molecular mechanisms. Int. J. Cardiol. Heart Vasc. *10*, 17–24. https://doi.org/10.1016/j.ijcha.2015.11.004.

Mordente, A., Meucci, E., Silvestrini, A., Martorana, G., and Giardina, B. (2009). New developments in anthracycline-induced cardiotoxicity. Curr. Med. Chem. *16*, 1656–1672. https://doi.org/10.2174/092986709788186228.

Oh, J.G., Kho, C., Hajjar, R.J., and Ishikawa, K. (2019). Experimental models of cardiac physiology and pathology. Heart Fail. Rev. *24*, 601–615. https://doi.org/10.1007/s10741-019-09769-2.

Onakpoya, I.J., Heneghan, C.J., and Aronson, J.K. (2016). Post-marketing withdrawal of 462 medicinal products because of adverse drug reactions: a systematic review of the world literature. BMC Med. *14*, 10. https://doi.org/10.1186/s12916-016-0553-2.

Onakpoya, I.J., Heneghan, C.J., and Aronson, J.K. (2018). Post-marketing withdrawal of analgesic medications because of adverse drug reactions: a systematic review. Expet Opin. Drug Saf. *17*, 63–72. https://doi.org/10.1080/14740338.2018.1398232.

Pound, P., and Ritskes-Hoitinga, M. (2018). Is it possible to overcome issues of external validity in preclinical animal research? Why most animal models are bound to fail. J. Transl. Med. *16*, 304. https://doi.org/10.1186/s12967-018-1678-1.

Pugazhendhi, A., Edison, T.N.J.I., Velmurugan, B.K., Jacob, J.A., and Karuppusamy, I. (2018). Toxicity of Doxorubicin (Dox) to different experimental organ systems. Life Sci. *200*, 26–30. https://doi.org/10.1016/j.lfs.2018.03.023.

Renu, K., V, G.A., P, B.T., and Arunachalam, S. (2018). Molecular mechanism of doxorubicin-induced cardiomyopathy - An update. Eur. J. Pharmacol. *818*, 241–253. https://doi.org/10.1016/j.ejphar.2017.10.043.

Ribeiro, M.C., Rivera-Arbeláez, J.M., Cofiño-Fabres, C., Schwach, V., Slaats, R.H., Ten Den, S.A., Vermeul, K., van den Berg, A., Pérez-Pomares, J.M., Segerink, L.I., et al. (2022). A New Versatile Platform for Assessment of Improved Cardiac Performance in Human-Engineered Heart Tissues. J. Personalized Med. *12*, 214. https://doi.org/10.3390/jpm12020214.

Richards, D.J., Li, Y., Kerr, C.M., Yao, J., Beeson, G.C., Coyle, R.C., Chen, X., Jia, J., Damon, B., Wilson, R., et al. (2020). Human cardiac organoids for the modelling of myocardial infarction and drug cardiotoxicity. Nat. Biomed. Eng. *4*, 446–462. https://doi.org/10.1038/s41551-020-0539-4.

Rivera-Arbeláez, J.M., Cofiño-Fabres, C., Schwach, V., Boonen, T., Ten Den, S.A., Vermeul, K., van den Berg, A., Segerink, L.I., Ribeiro, M.C., and Passier, R. (2022). Contractility analysis of human engineered 3D heart tissues by an automatic tracking technique using a

standalone application. PLoS One *17*, e0266834. https://doi.org/10.1371/journal.pone.0266834.

Robinson, E.L., Ameri, P., Delrue, L., Vanderheyden, M., Bartunek, J., Altieri, P., Heymans, S., and Heggermont, W.A. (2023). Differential expression of epigenetic modifiers in early and late cardiotoxic heart failure reveals DNA methylation as a key regulator of cardiotoxicity. Front. Cardiovasc. Med. *10*, 884174. https://doi.org/10.3389/fcvm.2023.884174.

Sawyer, D.B. (2013). Anthracyclines and heart failure. N. Engl. J. Med. Overseas. Ed. *368*, 1154–1156. https://doi.org/10.1056/NEJMcibr1214975.

Schlander, M., Hernandez-Villafuerte, K., Cheng, C.Y., Mestre-Ferrandiz, J., and Baumann, M. (2021). How Much Does It Cost to Research and Develop a New Drug? A Systematic Review and Assessment. Pharmacoeconomics *39*, 1243–1269. https://doi.org/10.1007/s40273-021-01065-y.

Schwach, V., Slaats, R.H., Cofiño-Fabres, C., Ten Den, S.A., Rivera-Arbeláez, J.M., Dannenberg, M., van Boheemen, C., Ribeiro, M.C., van der Zanden, S.Y., Nollet, E.E., et al. (2024). A safety screening platform for individualized cardiotoxicity assessment. iScience *27*, 109139. https://doi.org/10.1016/j.isci.2024.109139.

Shin, H.S., Shin, H.H., and Shudo, Y. (2021). Current Status and Limitations of Myocardial Infarction Large Animal Models in Cardiovascular Translational Research. Front. Bioeng. Biotechnol. *9*, 673683. https://doi.org/10.3389/fbioe.2021.673683.

Singal, P.K., and Iliskovic, N. (1998). Doxorubicin-induced cardiomyopathy. N. Engl. J. Med. Overseas. Ed. *339*, 900–905. https://doi.org/10.1056/nejm199809243391307.

Singh, G., Mémoli, F., and Carlsson, G.E. (2007). In Topological Methods for the Analysis of High Dimensional Data Sets and 3D Object Recognition, M. Botsch, R. Pajarola, B. Chen, and M. Zwicker, eds. (The Eurographics Association), pp. 91–100.

Skaf, Y., and Laubenbacher, R. (2022). Topological data analysis in biomedicine: A review. J. Biomed. Inf. *130*, 104082. https://doi.org/10.1016/j.jbi.2022.104082.

Dodge, Y. (2008). Spearman Rank Correlation Coefficient. In The Concise Encyclopedia of Statistics (Springer), pp. 502–505. https://doi.org/10.1007/978-0-387-32833-1_379.

Sritharan, S., and Sivalingam, N. (2021). A comprehensive review on time-tested anticancer drug doxorubicin. Life Sci. *278*, 119527. https://doi.org/10.1016/j.lfs.2021.119527.

Stamm, P., Kirmes, I., Palmer, A., Molitor, M., Kvandova, M., Kalinovic, S., Mihalikova, D., Reid, G., Wenzel, P., Münzel, T., et al. (2021). Doxorubicin induces wide-spread transcriptional changes in the myocardium of hearts distinguishing between mice with preserved and impaired cardiac function. Life Sci. *284*, 119879. https://doi.org/10.1016/j.lfs.2021.119879.

Strigun, A., Wahrheit, J., Niklas, J., Heinzle, E., and Noor, F. (2012). Doxorubicin increases oxidative metabolism in HL-1 cardiomyocytes as shown by 13C metabolic flux analysis. Toxicol. Sci. *125*, 595–606. https://doi.org/10.1093/toxsci/kfr298.

Subramanian, I., Verma, S., Kumar, S., Jere, A., and Anamika, K. (2020). Multi-omics Data Integration, Interpretation, and Its Application. Bioinform. Biol. Insights *14*, 117793221989905. https://doi.org/10.1177/1177932219899051.

Sun, D., Gao, W., Hu, H., and Zhou, S. (2022). Why 90% of clinical drug development fails and how to improve it? Acta Pharm. Sin. B *12*, 3049–3062. https://doi.org/10.1016/j.apsb.2022.02.002.

Tan, M., Yin, Y., Ma, X., Zhang, J., Pan, W., Tan, M., Zhao, Y., Yang, T., Jiang, T., and Li, H. (2023). Glutathione system enhancement for cardiac protection: pharmacological options against oxidative stress and ferroptosis. Cell Death Dis. *14*, 131. https://doi.org/10.1038/s41419-023-05645-y.

Tauzin, G., Lupo, U., Tunstall, L., Pérez, J.B., Caorsi, M., Medina-Mardones, A.M., Dassatti, A., and Hess, K. (2021). giotto-tda: A topological data analysis toolkit for machine learning and data exploration. J. Mach. Learn. Res. *22*, 1–6.

Thiese, M.S., Arnold, Z.C., and Walker, S.D. (2015). The misuse and abuse of statistics in biomedical research. Biochem. Med. *25*, 5–11. https://doi.org/10.11613/bm.2015.001.

Tokarska-Schlattner, M., Lucchinetti, E., Zaugg, M., Kay, L., Gratia, S., Guzun, R., Saks, V., and Schlattner, U. (2010). Early effects of doxorubicin in perfused heart: transcriptional profiling reveals inhibition of cellular stress response genes. Am. J. Physiol. Regul. Integr. Comp. Physiol. *298*, R1075–R1088. https://doi.org/10.1152/ajpregu.00360.2009.

Van Norman, G.A. (2019). Limitations of Animal Studies for Predicting Toxicity in Clinical Trials: Is it Time to Rethink Our Current Approach? JACC Basic Transl. Sci. *4*, 845–854. https://doi.org/10.1016/j.jacbts.2019.10.008.

van Scherpenzeel, M., Conte, F., Büll, C., Ashikov, A., Hermans, E., Willems, A., van Tol, W., Kragt, E., Noga, M., Moret, E.E., et al. (2022). Dynamic tracing of sugar metabolism reveals the mechanisms of action of synthetic sugar analogs. Glycobiology *32*, 239–250. https://doi.org/10.1093/glycob/cwab106.

Virshup, I., Rybakov, S., Theis, F.J., Angerer, P., and Wolf, F.A. (2021). anndata: Annotated data. Preprint at bioRxiv. https://doi.org/10.1101/2021.12.16.473007.

Voigt, N., Pearman, C.M., Dobrev, D., and Dibb, K.M. (2015). Methods for isolating atrial cells from large mammals and humans. J. Mol. Cell. Cardiol. *86*, 187–198. https://doi.org/10.1016/j.yjmcc.2015.07.006.

Volkova, M., and Russell, R., 3rd. (2011). Anthracycline cardiotoxicity: prevalence, pathogenesis and treatment. Curr. Cardiol. Rev. *7*, 214–220. https://doi.org/10.2174/157340311799960645.

Vriend, J., Hoogstraten, C.A., Venrooij, K.R., van den Berge, B.T., Govers, L.P., van Rooij, A., Huigen, M.C.D.G., Schirris, T.J.J., Russel, F.G.M., Masereeuw, R., and Wilmer, M.J. (2019). Organic anion transporters 1 and 3 influence cellular energy metabolism in renal proximal tubule cells. Biol. Chem. *400*, 1347–1358. https://doi.org/10.1515/hsz-2018-0446.

Warpe, V.S., Mali, V.R., S, A., Bodhankar, S.L., and Mahadik, K.R. (2015). Cardioprotective effect of ellagic acid on doxorubicin induced cardiotoxicity in wistar rats. J. Acute Med. *5*, 1–8. https://doi.org/10.1016/j.jacme.2015.02.003.

Weenen, H., Van Maanen, J.M.S., de Planque, M.M., McVie, J.G., and Pinedo, H.M. (1984). Metabolism of 4′-modified analogs of doxorubicin. unique glucuronidation pathway for 4′-epidoxorubicin. Eur. J. Cancer Clin. Oncol. *20*, 919–926. https://doi.org/10.1016/0277-5379(84)90165-2.

Weinstein, D.M., Mihm, M.J., and Bauer, J.A. (2000). Cardiac peroxynitrite formation and left ventricular dysfunction following doxorubicin treatment in mice. J. Pharmacol. Exp. Therapeut. *294*, 396–401.

Wouters, O.J., McKee, M., and Luyten, J. (2020). Estimated Research and Development Investment Needed to Bring a New Medicine to Market, 2009-2018. JAMA *323*, 844–853. https://doi.org/10.1001/jama.2020.1166.

Yuan, Y., Fan, S., Shu, L., Huang, W., Xie, L., Bi, C., Yu, H., Wang, Y., and Li, Y. (2020). Exploration the Mechanism of Doxorubicin-Induced Heart Failure in Rats by Integration of Proteomics and Metabolomics Data. Front. Pharmacol. *11*, 600561. https://doi.org/10.3389/fphar.2020.600561.

Zamorano, J.L., Lancellotti, P., Rodriguez Muñoz, D., Aboyans, V., Asteggiano, R., Galderisi, M., Habib, G., Lenihan, D.J., Lip, G.Y.H., Lyon, A.R., et al. (2016). 2016 ESC Position Paper on cancer treatments and cardiovascular toxicity developed under the auspices of the ESC Committee for Practice Guidelines: The Task Force for cancer treatments and cardiovascular toxicity of the European Society of Cardiology (ESC). Eur. Heart J. *37*, 2768–2801. https://doi.org/10.1093/eurheartj/ehw211.

Zaragoza, C., Gomez-Guerrero, C., Martin-Ventura, J.L., Blanco-Colio, L., Lavin, B., Mallavia, B., Tarin, C., Mas, S., Ortiz, A., and Egido, J. (2011). Animal models of cardiovascular diseases. BioMed Res. Int. *2011*, 497841. https://doi.org/10.1155/2011/497841.

Zhang, J., Cui, L., Han, X., Zhang, Y., Zhang, X., Chu, X., Zhang, F., Zhang, Y., and Chu, L. (2017). Protective effects of tannic acid on acute doxorubicin-induced cardiotoxicity: Involvement of suppression in oxidative stress, inflammation, and apoptosis. Biomed. Pharmacother. *93*, 1253–1260. https://doi.org/10.1016/j.biopha.2017.07.051.

Zhou, B., Shi, X., Tang, X., Zhao, Q., Wang, L., Yao, F., Hou, Y., Wang, X., Feng, W., Wang, L., et al. (2022). Functional isolation, culture and cryopreservation of adult human primary cardiomyocytes. Signal Transduct. Target. Ther. *7*, 254. https://doi.org/10.1038/s41392-022-01044-5.

Zhu, C., Wang, Y., Liu, H., Mu, H., Lu, Y., Zhang, J., and Huang, J. (2017). Oral administration of Ginsenoside Rg1 prevents cardiac toxicity induced by doxorubicin in mice through anti-apoptosis. Oncotarget *8*, 83792–83801. https://doi.org/10.18632/oncotarget.19698.

Stem Cell Reports, Volume *21*

Supplemental Information

Cross-modal integration of metabolomics and cardiac functionality captures dynamic metabotoxic effects of doxorubicin in engineered heart tissues

Federica Conte, Doroteya K. Staykova, Carla Cofiño-Fabres, Danique Snippert, Arno van Rooij, Dirk J. Lefeber, and Robert Passier

Supplemental Data Items

Cross-modal Integration of Metabolomics and Cardiac Functionality Captures Dynamic Metabotoxic Effects of Doxorubicin in Engineered Heart Tissues

Conte, Staykova *et al.* (2025)

Supplemental Items

- **Supplemental Text S1** (page 3): Node enrichment benchmarking.
- **Figure S1** (page 4): 3D-EHT after incubation in metabolite extraction buffer.

  Related to paragraphs "*On-pillar metabolite extraction for sequential functional and metabolic assessment of 3D-EHTs*" and "*On-pillar extraction of polar metabolites*"
- **Figure S2** (page 5): Optic microscope pictures of the 3D-EHTs treated with doxorubicin.

  Related to paragraph "*Doxorubicin reduces contraction force and alters CCM in 3D-EHTs*" and *Figure 2*
- **Figure S3** (page 6): Unfiltered hierarchical cluster plot.

  Related to paragraph "*Node enrichment provides a quantitative measure of association of metabolites with FoC and treatment*" and *Figure 5*
- **Figure S4** (page 7): Significance analysis via standard statistics of TEA-based metabolomics dataset (Z-score boxplots).

  Related to paragraph "*Metabolomics data processing*"
- **Figure S5** (page 8): Significance analysis via standard statistics of TBA-based metabolomics dataset (Z-score boxplots).

  Related to paragraph "*Metabolomics data processing*"
- **Figure S6** (page 9): Significance analysis via standard statistics of AA-based metabolomics dataset (Z-score boxplots).

  Related to paragraph "*Metabolomics data processing*"

Supplemental data

The following supplemental files (1-4) and the original code have been deposited at deposited at FigShare.com, under the DOI: https://doi.org/10.6084/m9.figshare.27985364.v2, and are publicly available at as of the date of publication.

- **Supplemental File S1**: List of MS transitions of target metabolites used for data acquisition.

- **Supplemental File S2**: Samples' metabolite levels (normalized and Z-transformed), dsDNA content and metadata.
- **Supplemental File S3**: Interactive topological connectivity network (TCN).
- **Supplemental File S4**: Data matrix with node values across the entire TCN used for correlation analysis.
- **Original data processing code (Python)** used for TDA-guided integration and visualization.

Raw data files have been deposited in the FAIR-compliant repository MetaboLights, under the accession number MTBLS13225, and are publicly available as of the date of publication.

Supplemental Text S1. Node enrichment benchmarking

After constructing the TCN, sample subsets from each node were extracted. Nodes sharing edges contained overlapping samples. For each cluster, mean metabolite values were computed and defined as node values (or coordinates) in the metabolic space. For categorical metadata (e.g., treatment, timepoint, or their combination), node values represented the percentage of samples with the respective category. These node values were assembled into a new matrix spanning the entire network (**Supplemental File 4**), from which a Spearman rank correlation matrix was calculated to estimate associations between data and metadata.

To benchmark our de novo method for inferring relationships of interest via node-based calculations, we evaluated the correlations between FoC and the metabolites. Being a numerical-valued parameter, FoC enabled the estimation of Spearman's rank correlation coefficients in two ways, namely (i) via the Z-scores and (ii) via the node values associated with each metabolite, referred as ground-truth and de novo correlations, respectively. Through plotting the latter as coordinates in a scatter plot, we visually compared the correlation coefficients obtained by both methods and estimated how the node-based coefficients approximated the ground-truth ones (**Figures 5A-D**).

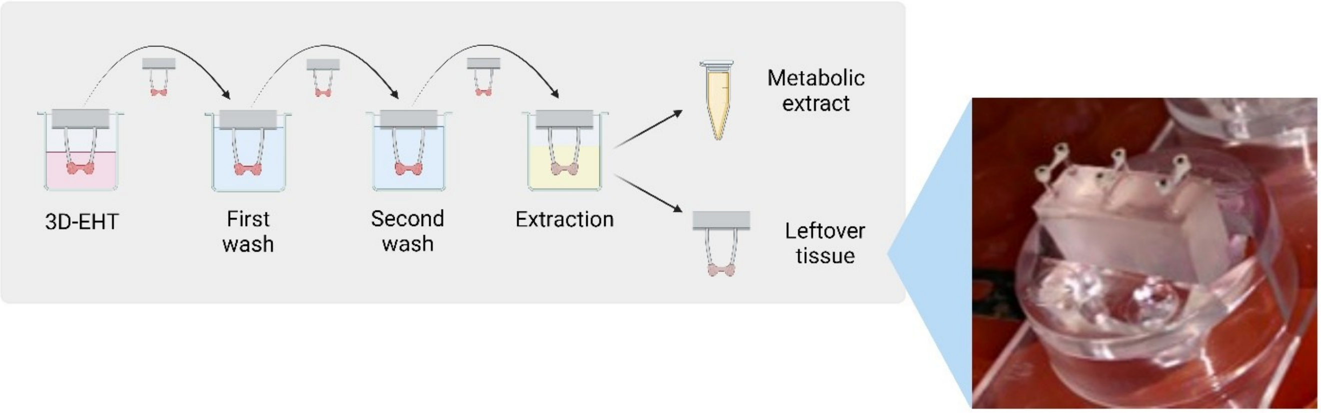

Figure S1.

3D-EHT tissues after 10 minutes incubation in organic extraction buffer during on-pillar extraction. With the correct handling, our extraction method ensures the preservation of the tissue integrity for later use (e.g. dsDNA extraction). Note: the picture illustrates a PDMS holder with three pairs of pillars that was used, however for our study, the design of the holder was

customized to one pillar set only, to enable on-pillar extraction from single tissues.

Treatment		*Replicate 1*	*Replicate 2*	*Replicate 3*
Untreated	0 hours	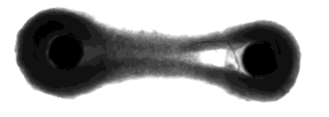	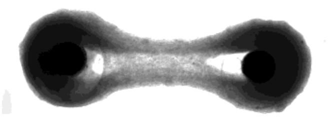	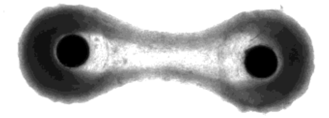
DMSO	24 hours	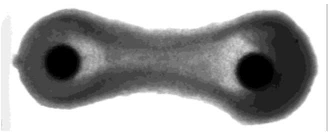	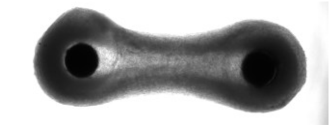	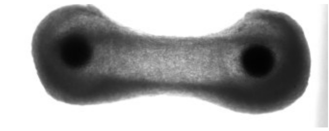
DOXO 1 uM	24 hours	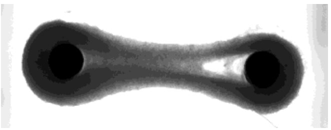	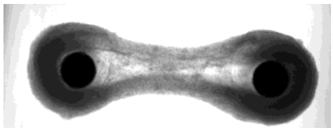	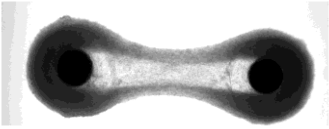
DOXO 5 uM	24 hours	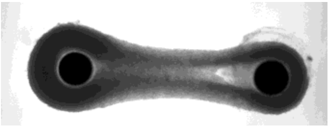	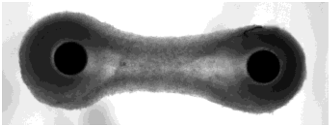	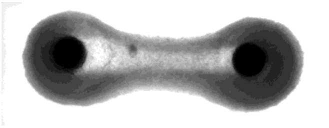
DMSO	48 hours	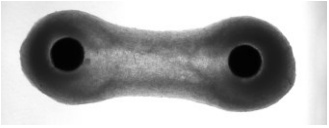	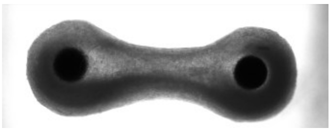	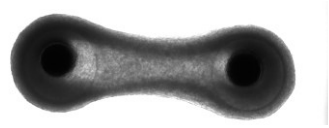
DOXO 1 uM	48 hours	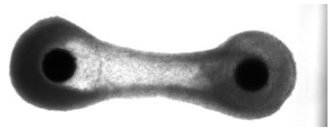	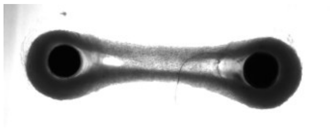	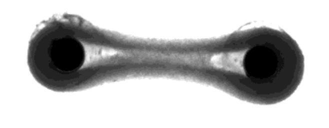
DOXO 5 uM	48 hours	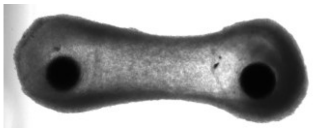	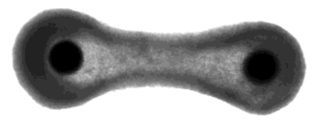	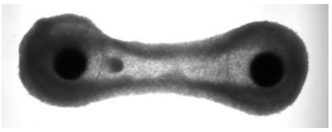

Figure S2.

Pictures of all 3D-EHT replicates acquired after electrical pacing and immediately before FoC measurement and metabolite extraction using a Nikon Ti2-E inverted microscope. The pictures were acquired when each tissue was at maximum extension (relaxed state). Distance between pillars' heads: 3 mm.

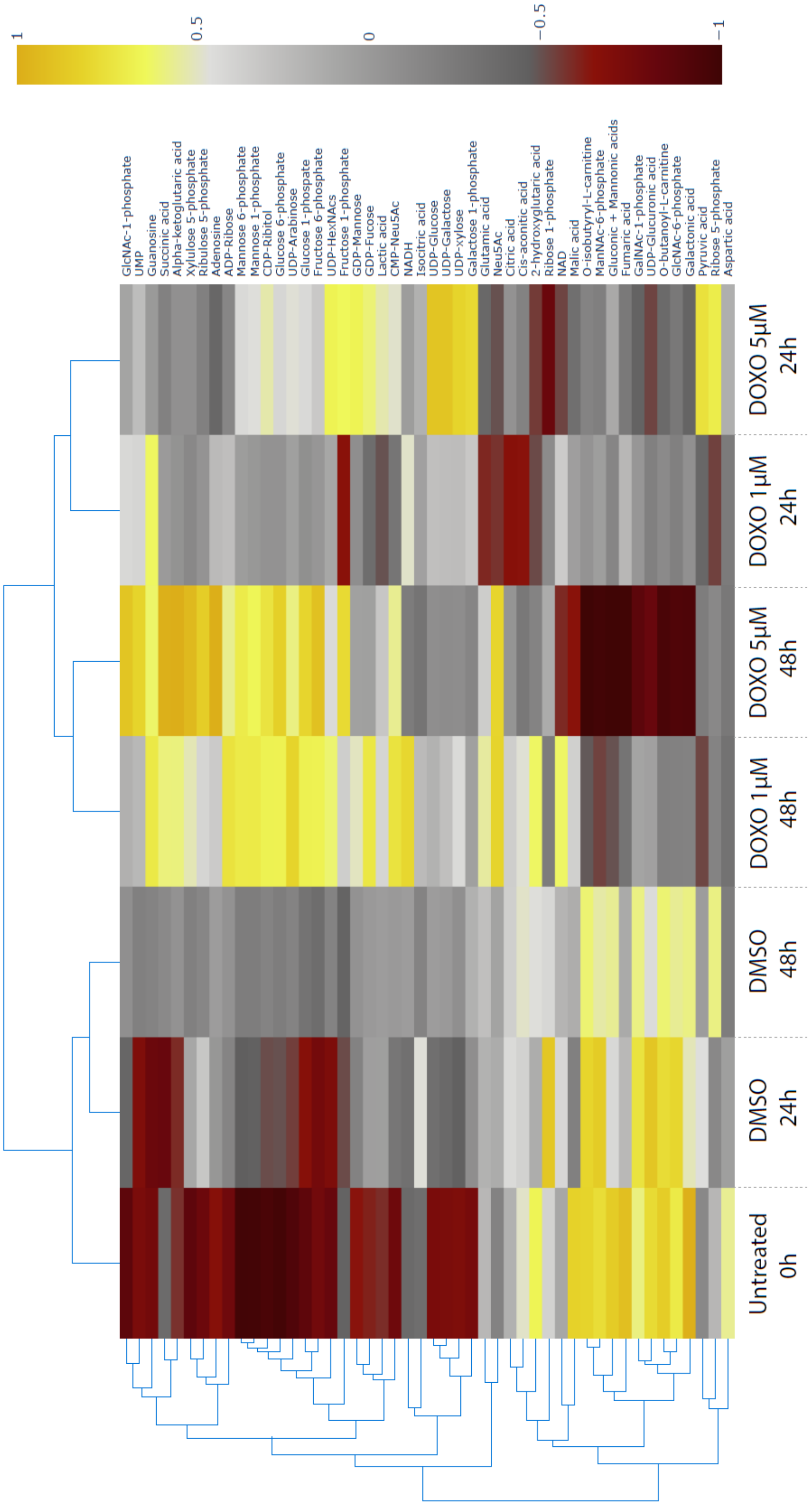

Figure S3.

Heatmap with dendrograms of hierarchical clustering on metabolites and treatment-time pairs based on their Spearman's rank correlation coefficients prior to filtering. For more details see main text.

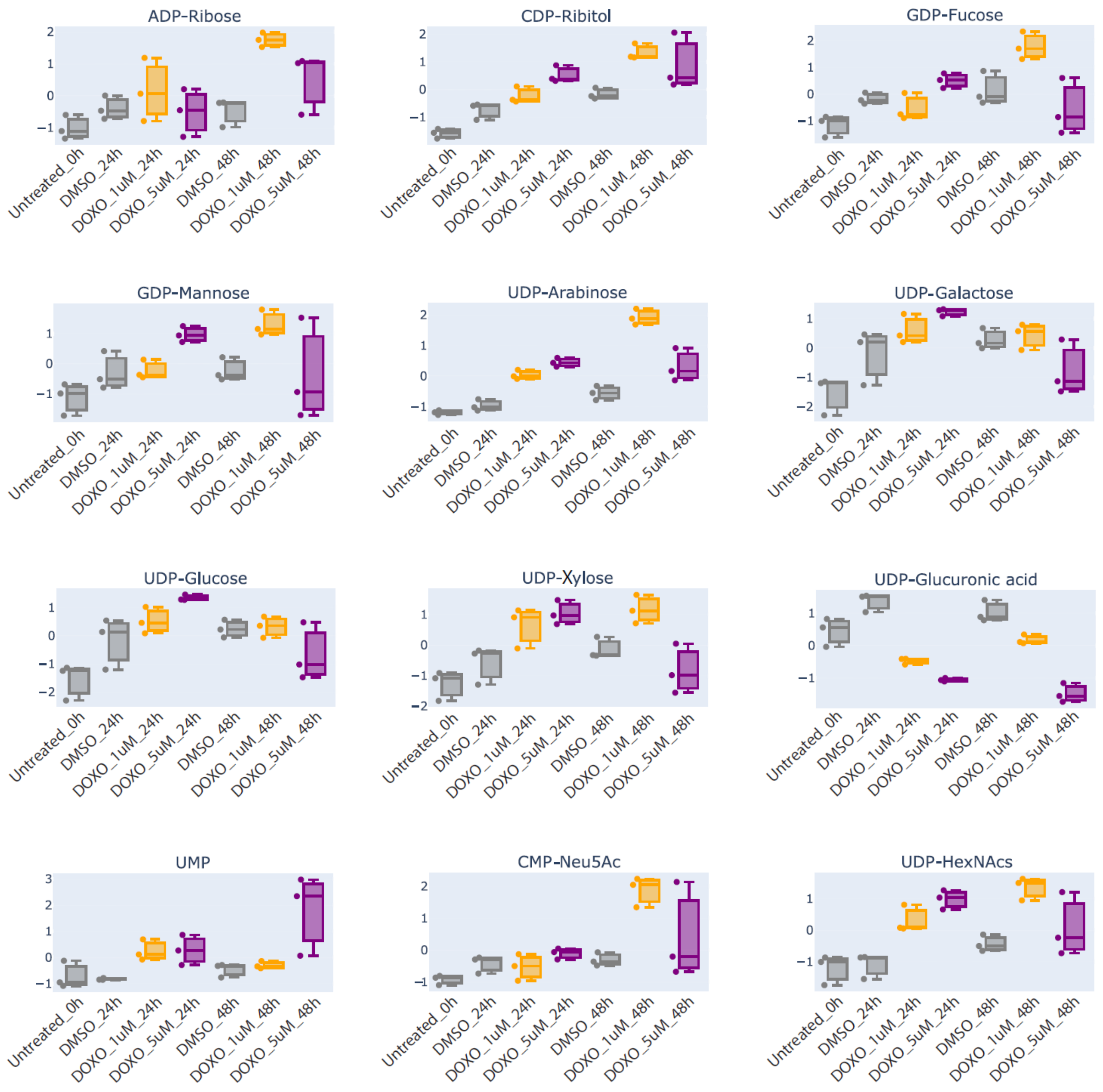

Figure S4.

Boxplots of the Z-transformed normalized levels of the metabolites detected via TEA-based MS method. Abbreviations: see main figures.

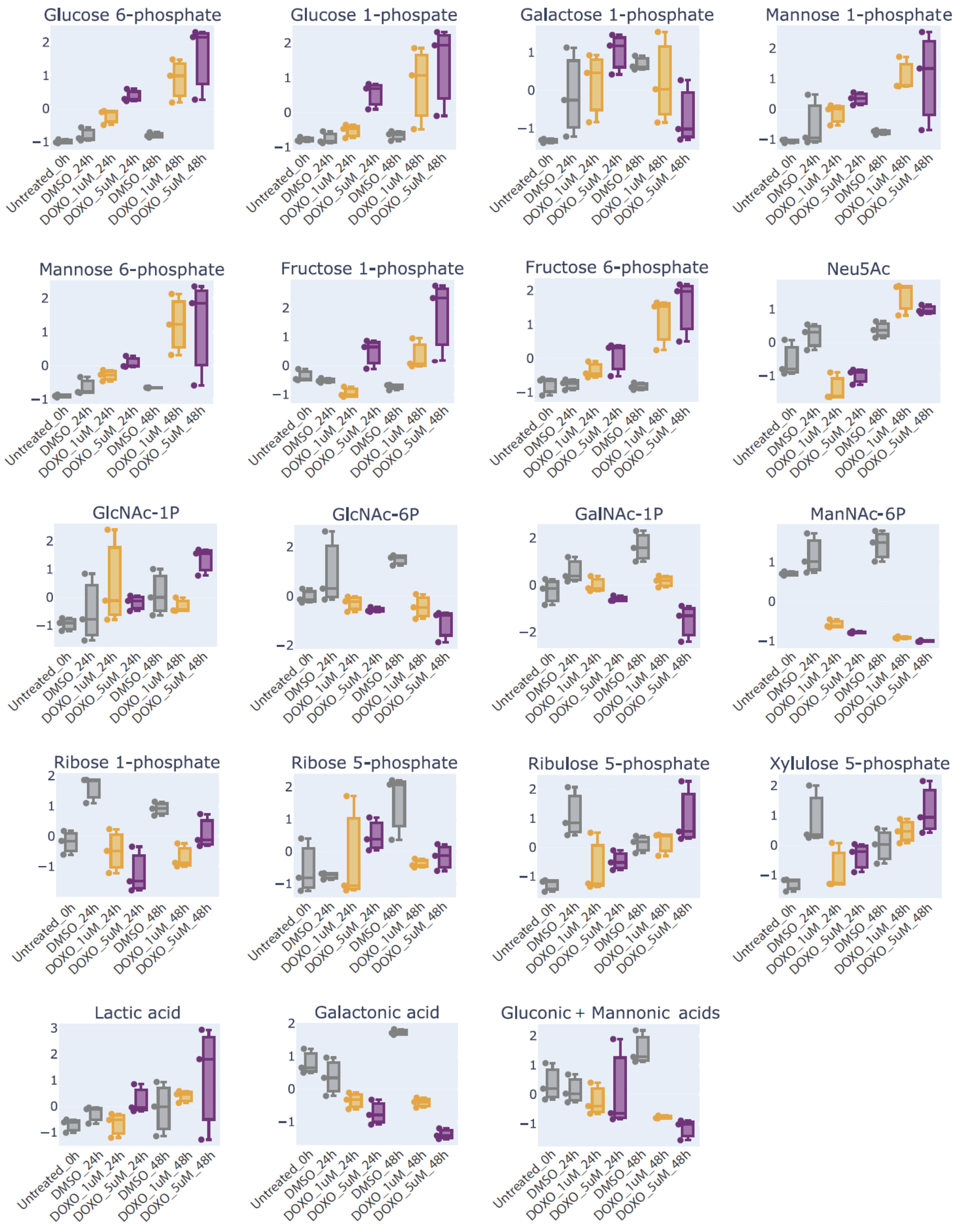

Figure S5.

Boxplots of the Z-transformed normalized levels of the metabolites detected via TBA-based MS method. Abbreviations: see main figures.

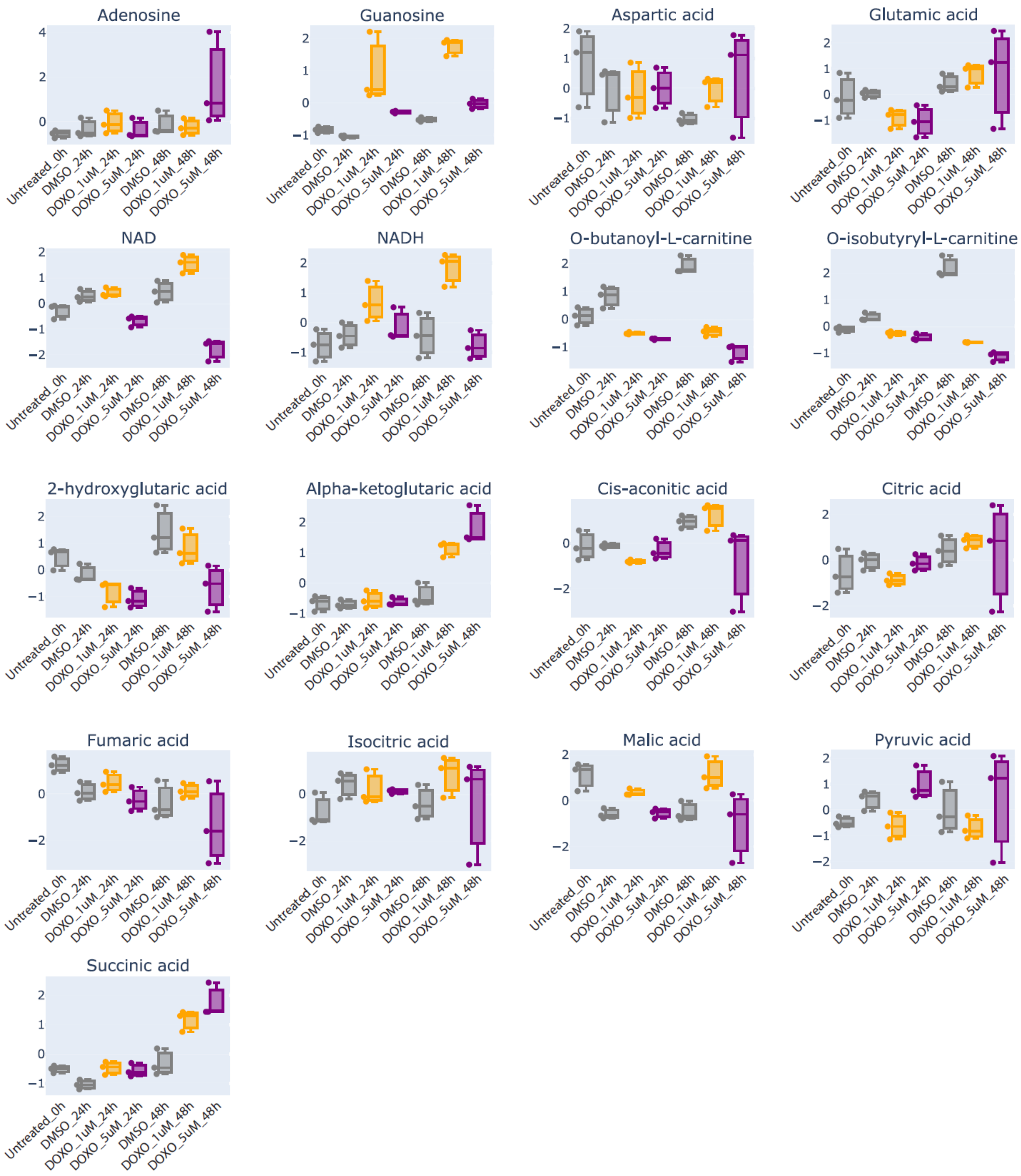

Figure S6.

Boxplots of the Z-transformed normalized levels of the metabolites detected via AA-based MS method. Abbreviations: see main figures.
